# Genome-wide analysis of chromosomal import patterns after natural transformation of *Helicobacter pylori*

**DOI:** 10.1038/ncomms11995

**Published:** 2016-06-22

**Authors:** Sebastian Bubendorfer, Juliane Krebes, Ines Yang, Elias Hage, Thomas F. Schulz, Christelle Bahlawane, Xavier Didelot, Sebastian Suerbaum

**Affiliations:** 1 Institute of Medical Microbiology and Hospital Epidemiology, Hannover Medical School, Carl-Neuberg-Strasse 1, 30625 Hannover, Germany; 2 DZIF—German Center for Infection Research, Hannover-Braunschweig Site, Carl-Neuberg-Strasse 1, 30625 Hannover, Germany; 3 Institute of Virology, Hannover Medical School, Carl-Neuberg-Strasse 1, 30625 Hannover, Germany; 4 Department of Infectious Disease Epidemiology, Imperial College, Norfolk Place, London W2 1PG, UK

## Abstract

Recombination plays a dominant role in the evolution of the bacterial pathogen *Helicobacter pylori*, but its dynamics remain incompletely understood. Here we use an *in vitro* transformation system combined with genome sequencing to study chromosomal integration patterns after natural transformation. A single transformation cycle results in up to 21 imports, and repeated transformations generate a maximum of 92 imports (8% sequence replacement). Import lengths show a bimodal distribution with averages of 28 and 1,645 bp. Reanalysis of paired *H. pylori* genomes from chronically infected people demonstrates the same bimodal import pattern *in vivo*. Restriction endonucleases (REases) of the recipient bacteria fail to inhibit integration of homeologous DNA, independently of methylation. In contrast, REases limit the import of heterologous DNA. We conclude that restriction-modification systems inhibit the genomic integration of novel sequences, while they pose no barrier to homeologous recombination, which reconciles the observed stability of the *H. pylori* gene content and its highly recombinational population structure.

## Abstract (web summary)

Uptake and integration of exogenous DNA into the bacterial genome play an important role in the evolution of the pathogen *Helicobacter pylori*. Here, the authors describe a bimodal pattern of chromosomal integration and show how restriction-modification systems limit the import of heterologous DNA.

## Graphical abstract

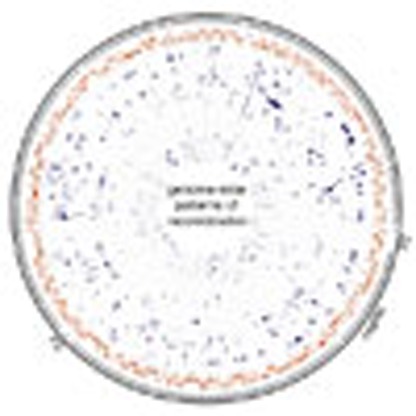

H*elicobacter pylori* is one of the most successful bacterial pathogens, infecting the stomachs of more than half of all humans. *H. pylori* infection causes chronic active gastritis which can progress to severe diseases, including gastroduodenal ulcers and gastric cancer^1^. The species displays exceptional genetic diversity and variability, partly due to a very high mutation rate^2^ that results from a lack of genes encoding a classical methyl-directed mismatch repair (MMR) system (*mutHLS1*)^3^,^4^ and from translesion-synthesis properties of its DNA polymerase I (ref. 5). An exceptionally high recombination rate is an additional hallmark of *H. pylori* and leads to the exchange of large proportions of its genome during mixed infections with different strains within one stomach^6^,^7^,^8^,^9^,^10^,^11^,^12^,^13^,^14^,^15^. Recombination has been shown to be the dominant driving force of genetic diversification of *H. pylori*, and introduces up to 109 times more substitutions than mutation^14^.

*H. pylori* is naturally competent for DNA uptake. Double-stranded DNA (dsDNA) is imported into the periplasm via the ComB transport system^16^,^17^, which is thought to be followed by conversion of dsDNA to single-stranded DNA (ssDNA) and subsequent transfer into the cytoplasm^18^. Within the cytoplasm, ssDNA interacts with DprA and RecA prior to recombination into the recipient's genome^19^,^20^. *In vitro*, mean import lengths depend on donor–recipient combinations, and range from 1.3 to 3.8 kb (refs 21, 22), whereas shorter imports were calculated from comparative sequence analyses of sequential patient isolates (estimated mean lengths: ∼400 bp)^6^,^9^,^10^.

*H. pylori* is unusually rich in restriction-modification (R-M) systems, which generally consist of a methyltransferase (MTase) activity targeting a distinct nucleotide sequence motif, and a cognate restriction endonuclease (REase) that can cleave the same motif when unmethylated. Out of 10 and 16 Type II R-M systems detected in the two best-studied *H. pylori* strains, 26695 and J99, only four REases are active while in the remaining systems, only the MTase remains as an active component^23^,^24^. It is unknown why R-M systems are so abundant in this pathogen, but recent studies have proposed that they play a role in limiting lateral gene transfer between *H. pylori* strains^25^,^26^.

Here we perform *in vitro* transformation experiments and analyse recombinant clones by whole-genome sequencing. We use defined donor–recipient combinations to elucidate genome evolution in *H. pylori* after single transformations or repeated transformation cycles (TCs). The data show a bimodal distribution of import lengths strongly suggesting two mechanisms of chromosomal integration of exogenous DNA. Our experiments also demonstrate that R-M systems limit the import of heterologous DNA, but do not provide a barrier against allelic exchanges of fragments of homeologous DNA. This is consistent with the observed high frequency of allelic replacements in the context of an overall highly conserved genomic content within the species *H. pylori*.

## Results

### Genome-wide patterns of DNA import after transformation

To perform a genome-wide analysis of chromosomal import patterns after natural transformation of *H. pylori*, two different transformation protocols were used. The first involved multiple cycles of transformation with the aim to increase the likelihood that recombinant clones contained many imports. The second protocol characterized recombinant clones obtained by one single transformation. *H. pylori* strain 26695 (ref. 3) was used as a recipient in transformations with purified genomic DNA (gDNA) extracted from the donor strain J99-R3 *rdxA*::CAT *flaA*::*aphA-3* (J99-R3^Cm^). This strain is a derivative of *H. pylori* strain J99 (ref. 4) which contains a point mutation in the *rpoB* locus conferring resistance to rifampicin^21^ plus two resistance cassettes encoding chloramphenicol and kanamycin resistance in non-essential genes, *rdxA* and *flaA*, respectively. The published genome sequences of strains 26695 and J99 differ by ∼4.5% of nucleotides, such that, on average, one polymorphism occurs every 22 bp. This high frequency of sequence differences between donor and recipient allows a precise localization of imported sequences and their boundaries within the recombinant genomes.

The repeated transformation experiments (TC) were performed for 28–52 days (Supplementary Fig. 1). Four independent recipient cultures were grown overnight (14 h) before donor DNA was added and the culture incubated for another 10 h. After this, an aliquot was transferred to fresh medium and a new overnight incubation started (see Methods for details). In parallel, a control culture was incubated without addition of donor DNA. After completion of the final TC, cells were plated onto non-selective blood-agar plates and single colonies were isolated. A total of 20 clones from cultures transformed with donor DNA and 2 clones from control cultures without DNA addition were randomly chosen and subjected to whole-genome sequencing.

For the short-term transformation (STT) experiments, we performed one single round of transformation, using a large excess of donor DNA (50 donor genomes per recipient cell; Supplementary Fig. 1). Single colonies were first isolated from non-selective agar. Despite the large amount of DNA used for transformation, genome-sequence comparisons of 10 randomly chosen clones from 2 independent experiments did not identify any recombination events. Thus, in contrast to the TC experiments, the STT experiments were performed under selection for clones that had acquired resistance to chloramphenicol. In total, the genomes of 21 clones—selected for the integration of the CAT cassette into the *rdxA* locus and derived from 2 independent experiments—were sequenced.

### Genome-wide distribution of recombination

**Figure 1:**
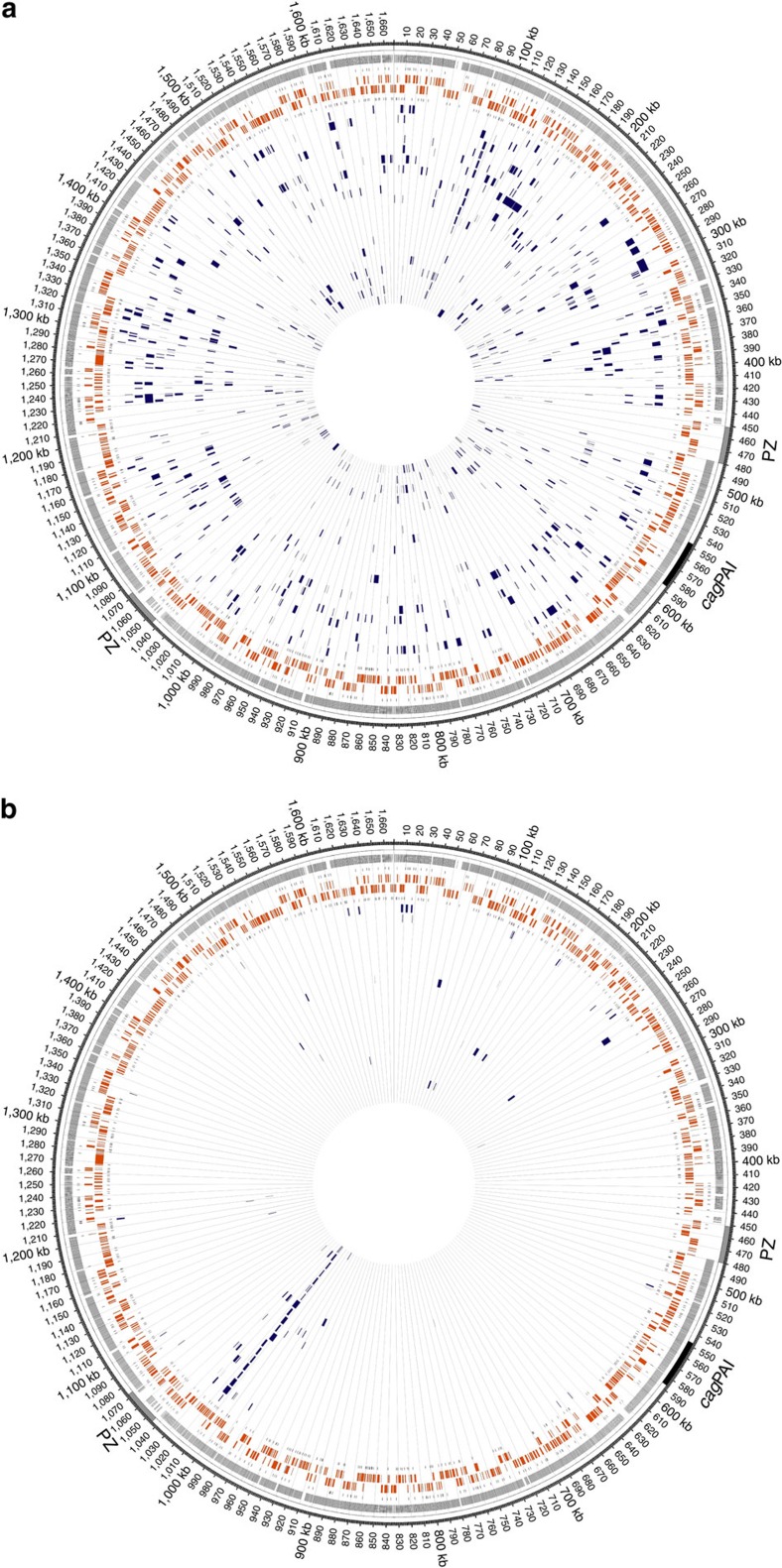
Circular representation of *H. pylori* 26695 recipient chromosome displaying SPIs and CNPs. The representations, generated using Circos^49^, display clusters of nucleotide polymorphisms (CNPs)/single-polymorphism imports (SPIs) in the genomes of recombinant clones obtained from the transformation cycle (**a**) and short-term transformation (**b**) experiments. Circles from outwards to inwards depict localization of plasticity zone (grey) and *cag* pathogenicity island (*cag*PAI), positions of sequence differences between donor strain and strain 26695, transcription-start sites on plus strand, ORFs on plus strand (orange), ORFs on minus strand (orange), transcription-start sites on minus strand and a variable number of genomes of transformed clones indicating the positions of imports. Grid marks distance of 10 kb. (**a**) Imports of 20 TC clones transformed with gDNA of J99-R3^Cm^ deriving from a total of four independent cultures (dark blue). (**b**) Imports of 21 STT clones transformed with gDNA of J99-R3^Cm^ deriving from a total of two independent cultures (dark blue). Position at ∼1,015 kb marks site of selected recombination (*rdxA*::CAT allele).

We next analysed the frequency and chromosomal distribution of import events (definitions provided in Methods and Supplementary Fig. 2) in clones from STT and TC experiments. As expected, not one single recombination event was detected in the clones derived from the control cultures without DNA addition. In contrast, all clones resulting from STT or TC experiments had imported one or multiple fragments of donor DNA (Fig. 1). Within 20 clones from the TC experiments, 826 imports were identified (mean, 41±24 imports per clone), replacing an average 3.3% of the recipient's genome (mean, 55.6±36.1 kb) (Table 1). For the STT experiment, 101 import events were identified in 21 sequenced clones. The large majority (∼80%) of these events represented non-selected imports. The STT clones contained a mean number of 4.8±4.5 imports per strain, introducing 6.7±5.2 kb of donor DNA into the recipient genome. Mean import lengths did not differ significantly between the TC and the STT experiments (1,347±1,587 versus 1,410±1,537 bp). In contrast, selected and non-selected imports differed significantly in length. Selected imports were significantly longer than non-selected imports (Welch's *t*-test: *P*<0.0001; Table 1) even after subtraction of the length of the cassette from the import length. This effect is likely a result of selection, since the resistance cassette must be integrated fully to express chloramphenicol resistance.

We further tested for evidence of selection for imports in individual functional gene categories. To this end, we assigned all genes affected by recombination to Clusters of Orthologous Groups of proteins (COGs)^27^,^28^ and, in addition, to the group of *hop* genes, which are part of the outer membrane protein (*omp*) family genes of *H. pylori*^29^. No significant overrepresentation of imports in any of these functional classes was detected in clones from the STT or TC experiments (Supplementary Fig. 3a,b), in contrast to the strong selection for clones with imports in *hop* genes previously observed during chronic *H. pylori* infection^9^,^15^. For the STT clones, a large part of the non-selected imports was located in the proximity of the selected recombination event, indicating linkage between selected imports and surrounding non-selected imports.

### Localization of *in vitro* recombination events

To better understand the distribution of recombination events within the recipient genomes, we first conducted a genome-wide analysis of the density of single nucleotide polymorphisms (SNPs) for the alignment of the 26695 and J99-R3^Cm^ genomes using a 200-bp sliding window (Supplementary Fig. 2b, upper right panel; Supplementary Fig. 4, upper panel). This was compared with the SNP density of 84 randomly selected authentic recombination events (SNP densities separately calculated for imported fragments and flanking sequences). We observed a similar SNP density in all three analyses, strongly indicating that import patterns are not determined by varying levels of similarity between homeologous recipient and donor fragments.

**Figure 2:**
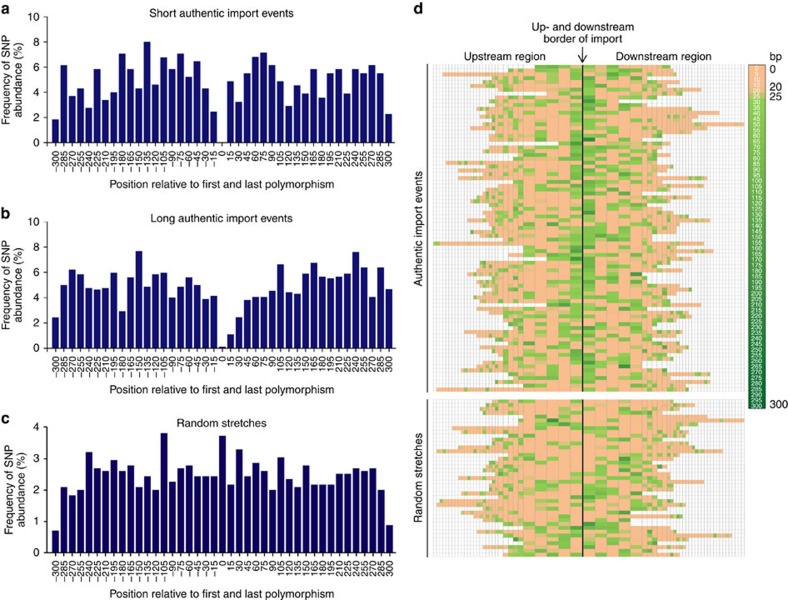
Graphical representation of SNP abundance and distances between SNPs near import borders. (**a**–**c**) Histograms depicting the relative SNP abundance within 15-bp bins in relation to the total number of SNPs identified within the flanking region of 300 bp upstream and downstream of 84 representative import events (24-short and 60-long import events) and 40 random stretches. The latter stretches were obtained by randomly assigning simulated 1-kb imports in ∼10-kb steps of the J99-R3^Cm^ donor genome and analysed regarding polymorphisms between donor and recipient sequence within the flanking region. Short authentic imports are <300 bp, while long authentic imports are >300 bp. The position 0 labels the first and the last position of the analysed events, thus, eliminating the size of the import or random stretch. (**d**) Heatmap visualizing the distances between SNPs for the same set of 84 representative import events and 40 random stretches in a window of 300 bp upstream and downstream of import/stretch borders. Import/stretch borders are indicated by a single vertical black line. For each import or random stretch, the distances between individual SNPs were calculated and a heatmap colour code was applied that marked short distances (0–19 bp) in rose and long distances (26–300 bp) with a light to dark green colour. Intermediate distances (20–25 bp) are marked in a rose-green mixture colour. Each box depicts the distance between two SNPs upstream or downstream of one individual import/stretch. The width of the boxes does not correlate with distances between SNPs. The boxes representing the distances between the first five SNPs proximal to import/stretch borders the widest, followed by five intermediate-sized boxes depicting the next five distance values, and a variable number of narrow boxes. Different numbers of coloured boxes are a consequence of different SNP abundances up- and downstream of individual imports/stretches. Non-coloured boxes mark regions that lie in the outer rim of the 300-bp window upstream or downstream of imports/stretches and are therefore not included into the heatmap colouring.

We next analysed whether recombination was favoured by the presence of longer stretches of identical sequence in the immediate proximity of import borders. Therefore, we extended the analysis to include information about the positions of differences between donor and recipient sequences within a fixed 300-bp window covering the flanking regions up- and downstream of imports. For this, the same set of 84 authentic imports was compared with 40 ‘simulated imports', that is, randomly assigned stretches of the donor genome with sizes of ∼1,000 bp, which resembles the median lengths of imports observed in this study. Like observed imports, simulated imports were selected such that they start and end with a SNP. We observed a strong decrease in the abundance of differences within a 30-bp region immediately up- and downstream of import borders, suggesting that the presence of a stretch of identical sequence strongly increases the likelihood of recombination (Fig. 2a,b). In contrast, the abundance of SNPs upstream and downstream of the random stretches was evenly distributed (Fig. 2c).

Next, we specifically analysed the regions directly flanking the imports for stretches of identical nucleotides. Using the same data set of 84 authentic imports, we plotted the distances between the SNP bordering an import and the first flanking donor-identical SNP that was not incorporated into the recipient clone's genome. This distance was compared with the values determined for the distance between the first and the second donor-identical SNP (Supplementary Fig. 5a). We detected a statistically significant difference between these two values (Welch's *t*-test), which was not observed for our control data set described above. The distribution of the distance to the first SNP upstream or downstream of recombination events showed a clear dearth of small values relative to a Geometric model, but fitted well with a Negative-Binomial model with parameter *r*=2 (Supplementary Fig. 5b). This would indicate that one less SNP is observed on either side of recombination events than expected if they were randomly distributed around the genome. This finding prompted us to search for a minimal requirement of identical sequence to facilitate recombination. To this end, we generated a heatmap based on the distances between differences flanking authentic imports and random stretches determined before. Identical sequence stretches of ≥25 bp were more common in the immediate vicinity of authentic imports (77%) versus randomly selected DNA fragments (35%) (Fig. 2d), indicating that short tracts of identical sequence facilitate recombination. However, no strict minimal identity criterion could be defined, because there was a substantial number of imports with SNPs within very few base pairs of the import border (Fig. 2d), indicating that the relationship between the presence of stretches of sequence identity and the placement of recombination endpoints is probabilistic.

### Bimodal distribution of import lengths

**Figure 3:**
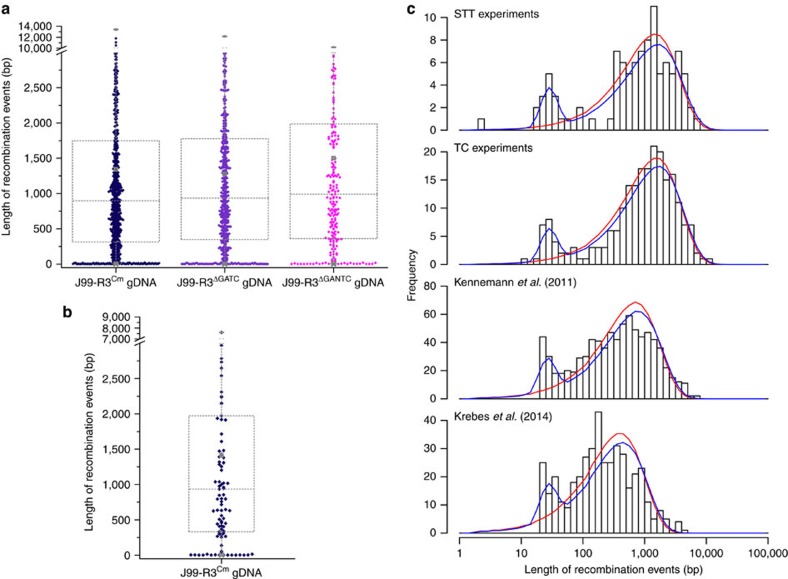
Box-whisker-plots representing the distribution of import lengths and graphical representation of micro- and macro-imports in *H. pylori*. (**a**,**b**) Individual lengths of all CNPs/SPIs from TC ((**a**) J99-R3^Cm^: 826 imports; J99-R3^ΔGATC^: 547 imports; J99-R3^ΔGANTC^: 224 imports) and STT ((**b**) J99-R3^Cm^: 101 imports) clones are indicated by diamonds. In the superimposed box and whisker plots, the arithmetic mean is labelled by a square, the 99% percentile by a cross and the maximum length by a horizontal line (all grey). Colour coding of TC imports: J99-R3^Cm^: dark blue; J99-R3^ΔGATC^: purple; J99-R3^ΔGANTC^: light purple. Colour coding of STT imports equals TC imports transformed with J99-R3^Cm^. (**c**) The length distribution of import events of the STT and TC clones was compared with *in vivo* data on recombination event lengths from recent studies. The *y*-axis indicates the number of events. The length distribution of import events was plotted on the *x*-axis using a logarithmic scale. The red and blue lines are the maximum-likelihood fit of the ‘simple' and ‘mixture' model, respectively.

Next, we sought to characterize the length distribution of imports in clones derived from the TC and the STT experiments. In addition to many imports with a size ∼1 kb, we observed a substantial number of very short imports, ranging between 1 and 50 bp (minimal length). When imports were plotted by using the average lengths of all imports for both the TC, as well as the STT data (Fig. 3a,b), a bimodal distribution of import lengths became apparent.

To confirm this observation and assess its statistical significance, we fitted two different models to the distribution of import lengths (Fig. 3c). The ‘simple' model assumes that the lengths of all imports are normally distributed. This model only has a single parameter, representing the mean length of all imports, which is 1,557 bp and 1,470 bp for the TC and the STT, respectively. The ‘mixture' model combines two distinct distributions of import sizes, and uses three parameters: one for each of the two distributions and one for their relative weight. Importantly, this mixture model fits the TC, as well as the STT data better than the first model even when accounting for its higher complexity (TC: ΔAICc=43, ΔBIC=37; STT: ΔAICc=27, ΔBIC=21). This result confirms that two populations of import sizes are present in *H. pylori*, one with a mean length of 28 bp (TC and STT), and one with mean lengths of 1,690 bp and 1,645 bp for TC and STT imports, respectively. We introduce the terms micro-import and macro-import for these two groups of imports. The proportion of recombination events originating from micro-imports was estimated to be 9% and 11% for the TC data and STT data, respectively.

### Bimodal import patterns in paired *in vivo* isolates

We next asked whether the observed bimodal distribution of import patterns was restricted to clones obtained by *in vitro* transformation, or whether a similar pattern can be observed after recombination that occurred *in vivo* during mixed infection of humans. We therefore reanalyzed recombination events within genomes of four pairs of sequential isolates with a distance of 3 years (NQ315/NQ1712, NQ352/NQ1701, NQ392/NQ1707 and NQ367/NQ1671) and two pairs with a distance of 16 years (NQ392/NQ4060 and NQ367/NQ4191) from chronically infected Colombian patients^9^ (Fig. 3c) and in addition, recombination events in genomes of two familial isolates (H3017 and H3018) from chronically infected individuals of a family from Coventry, UK^10^ (Fig. 3c). Importantly, the mixture model also fits best for these *in vivo* data^9^,^10^, demonstrating that micro- and macro-imports also occur during chronic infection.

### Restriction of DNA limits integration of heterologous sequences

**Figure 4:**
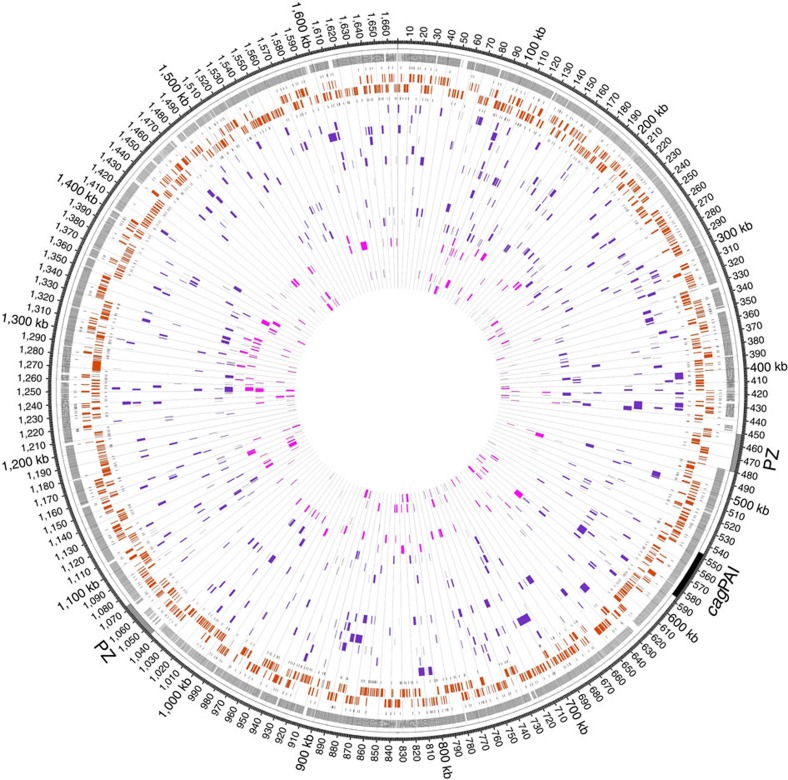
CNPs/SPIs in transformed clones obtained from the TC experiments with donor gDNAs of two different MTase-deficient mutants. Circles from outwards to inwards depict the localization of plasticity zone (grey) and *cag* pathogenicity island (*cag*PAI), positions of sequence differences between donor strain and strain 26695, transcription-start sites on plus strand, ORFs on plus strand (orange), ORFs on minus strand (orange), transcription-start sites on minus strand and a variable number of genomes of transformed clones indicating the positions of imports. Figure depicts imports of 12 TC clones deriving from 2 independent cultures transformed with gDNA of J99-R3^ΔGATC^ (purple) and imports of 6 TC clones deriving from 1 culture transformed with gDNA of J99-R3^ΔGANTC^ (light purple). Grid marks distance of 10 kb.

R-M systems have been reported to function as a barrier to horizontal gene transfer in *H. pylori* and many other bacteria (see review (ref. 30)). These studies were based either on the analysis of homologous and homeologous recombination by selecting for mutated alleles conferring antibiotic resistance, or on the investigation of heterologous-sequence insertions using antibiotic resistance cassettes for selection. In the present study, we employed the TC approach to analyse the impact of methylation and restriction on genome-wide homeologous recombination in the absence of selection pressure. For this purpose, we used two isogenic mutants of J99-R3 as donors that lacked the GATC or GANTC Type II MTase activity due to the introduction of a kanamycin-resistance marker into the jhp0085 or jhp1271 locus, respectively (J99-R3^ΔGATC^, J99-R3^ΔGANTC^)^24^. The corresponding GATC and GANTC REases are active in the 26695 recipient strain^31^, which we re-confirmed for the 26695 strain used in our laboratory by restriction digestion assays with cell-free extracts. The gDNA of both MTase mutants was thus susceptible to restriction by REases expressed by the recipient strain. After 28 cycles of transformation, 12 and 6 randomly chosen clones transformed with DNA lacking GATC and GANTC methylation, respectively, were whole-genome sequenced and examined for their import characteristics (Fig. 4). Transformation of 26695 with gDNA lacking GATC methylation resulted in an average import number of 46±26 with a mean import length of 1,293±1,370 bp. Transformation with gDNA lacking GANTC methylation resulted in average import numbers of 37±27 and a mean import size of 1,500±1,624 bp. These values were not significantly different from those of clones resulting from the transformation of 26695 with gDNA of J99-R3^Cm^, methylated at both recognition sites (import frequency: 41±24 imports per clone, mean import length: 1,347±1,587 bp) (Table 1).

We also analysed whether GATC and GANTC recognition sites were preferentially found in donor sequences flanking imported fragments, which would be expected if restriction by GATC or GANTC REases had a role in determining recombination endpoints. To this end, the positions of ∼60 randomly selected import events for each donor–recipient combination were mapped onto the respective donor genome sequences. We then measured the distances between the import border and the proximal GATC or GANTC site in the region flanking the imported sequences (Supplementary Fig. 6). There was no statistically significant increase of the abundance of recognition sites in close proximity to import borders (Welch's *t*-test), providing additional evidence that R-M systems do not affect the chromosomal integration of homeologous DNA in *H. pylori*.

**Figure 5:**
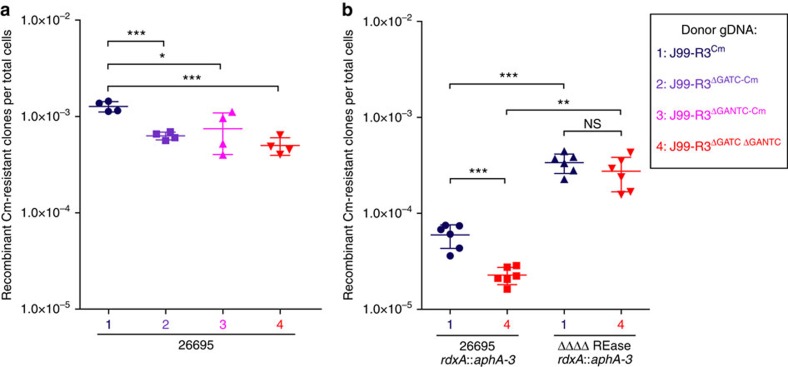
Effect of R-M systems on recombination of heterologous sequences in *H. pylori* 26695. Frequencies of recombinant chloramphenicol-resistant clones after natural transformation of 26695 wild-type (**a**) and 26695 ΔΔΔΔREase mutant (**b**) with different donor gDNAs. The ΔΔΔΔREase mutant is deficient in all four active Type II REases of *H. pylori* 26695. Legend displays colour coding of symbols. The arithmetic mean is indicated by a long vertical line and the s.d. by the error bars. The data were obtained from at least two independent experiments each performed in duplicates. Significant differences were determined using Welch's *t*-test (two-sided) and are labelled with asterisks (*P<0.05, **P<0.01, ***P<0.001). NS, not significant.

To study the effect of R-M systems on the insertion of heterologous sequences into the recipient genome, we adapted a previously described *in vitro* transformation model^21^. To select for resistance to chloramphenicol, a CAT cassette was introduced into the *rdxA* gene (*rdxA*::CAT) of both single MTase-deficient J99-R3 mutants (J99-R3^ΔGATC^ and J99-R3^ΔGANTC^) and of a double mutant (J99-R3^ΔGATC ΔGANTC^). This heterologous resistance cassette was flanked by homeologous stretches of the *rdxA* gene. The cassette also contains a single GATC and a single GANTC site, thus rendering the (unmethylated) fragment susceptible to restriction by recipient REases. In addition, we constructed a 26695 quadruple mutant strain lacking all four active Type II REase genes (HP0053, HP0091, HP1351 and HP1366) using a modified protocol of the MuGENT (multiplex genome editing by natural transformation) technology initially described for *Vibrio cholerae* by Dalia *et al*.^32^ (see Methods). Wild-type strain 26695 and the isogenic REase mutant were transformed with J99-R3^Cm^ gDNA or with gDNA from either of the J99-R3 MTase mutants, and the recombination frequencies of the different donor–recipient combinations were determined (Fig. 5). The efficiency of transformation with donor gDNA lacking both GATC and GANTC methylation was significantly reduced (Welch's *t*-test: *P* value=0.0004) (Fig. 5, left panel). Separate inactivation of either GATC or GANTC methylation also significantly lowered the recombination frequency (*P* values=0.0045 or 0.0485, respectively). The deletion of all active Type II REase genes resulted in a significantly increased recombination frequency (*P* values=0.0022–0.0003) that was independent of the presence of both GATC and GANTC methylation on the donor DNA (Fig. 5, right panel).

## Discussion

Our data show that *in vitro* and *in vivo* transformation of a recipient *H. pylori* strain with chromosomal DNA from a donor strain generates imports whose length follows a bimodal distribution. One class of imports is very short (peak length 28 bp), and the second class of imports is much longer (peak length 1,645 bp). A similar bimodal distribution of recombinational imports was recently observed for *Streptococcus pneumoniae*^33^. Their study and model development was based on genome-sequence comparisons between related pneumococcal clinical isolates belonging to the two lineages PMEN1 and CC180, and confirmed by reanalysis of genome data from *in vitro* transformants. For consistency with previous publications, we use the terms micro- and macro-imports for the two classes of events with shorter and longer lengths, respectively. We note that in the study by Mostowy *et al*.^33^, the term macro-recombination is used for imports that can include multiple fragments interrupted by stretches of identity (‘saltational recombination'), which in our terminology would be grouped imports^9^. In our analyses, macro-imports were much more common than micro-imports (89 versus 11%), and since, on average, a macro-import introduces ∼60 times more polymorphisms into the recipient chromosome than a micro-import, they are responsible for the bulk of genome diversification. We note that the frequency of micro-imports is likely to be underestimated, as our donor and recipient sequences differed only by about 4.5% of nucleotides and many short imports are therefore likely to leave no traces since donor and recipient sequences are identical.

In pneumococci, both micro- and macro-recombination have been suggested to reflect the activity of the MMR system^33^, which normally prevents the integration of DNA with base mismatches. MMR in pneumococci is thought to abort the extension of recombination tracts with homeologous DNA, giving rise to micro-recombination. MMR can become saturated when the number of polymorphic nucleotides within the transformed fragment(s) exceeds 150, permitting the integration of longer fragments^34^. It seems surprising that *H. pylori* displays a similar pattern of DNA imports as pneumococci, while it lacks genes encoding typical MMR proteins^3^,^35^. The mechanisms that are responsible for the observed pattern of import lengths in *H. pylori* are currently unknown. We observed that DNA in the vicinity of imports did not display a lower density of SNPs than other regions of the donor genome, indicating that homology differences between donor and recipient strains do not predetermine the distribution of imports. However, our data also show that stretches of sequence identity between donor and recipient are significantly overrepresented next to import borders. This is reminiscent of minimal efficient processing elements (MEPS) important for homologous recombination in *Escherichia coli*^36^, indicating that homologous strand cross over preferentially takes place within longer stretches of identity. Our data do not support a strict identity criterion for initiation of recombination, but rather suggest a probabilistic relationship between the presence of stretches of sequence identity and generation of a recombination endpoint.

The average import lengths determined in this study are similar to those reported in previous *in vitro* studies in *H. pylori*^21^,^22^, and in a study of 34 pairs of sequential *H. pylori* isolates that was based on an extended MLST approach including 78 gene fragments^37^. These values are significantly longer than those calculated from whole-genome comparisons between pairs of sequential patient isolates or strains after recent transmission^9^,^10^. Several factors may account for the difference: currently, <10 full genome comparisons are available as a basis for calculations of import length in sequential patient isolates, so that the lower values may reflect a particularity of specific mixed infections (for example, combination of donor and recipient strain). Alternatively, shorter import lengths may be the result of specific conditions in the *in vivo* environment (for example, due to DNA damage under mildly acidic conditions, or to regulation of components of the DNA uptake and repair machinery *in vivo*), or of the observation of changes over several years where micro-imports with ‘recipient'-type DNA could lead to fragmentation of initially contiguous imports.

Our data indicate that restriction by GATC- and GANTC-specific REases and protection against this by cognate MTases do not affect the integration of stretches of homologous DNA, while they provide a strong barrier against the incorporation of novel DNA flanked by homologous sequences. It has long been thought that R-M systems in *H. pylori* provide a barrier to the uptake not only of plasmid DNA^38^,^39^,^40^,^41^, but also of chromosomal DNA from closely related strains^25^,^26^. The very high number of R-M systems in *H. pylori* was thus in apparent conflict with its well-established highly recombinogenic population structure. However, the previous studies targeting R-M systems as potential barriers to inter-strain recombination did not differentiate between homologous/homeologous recombination and the insertion of heterologous sequences via homologous/homeologous recombination. All previous studies that used chromosomal DNA from non-isogenic strains to test the effect of R-M systems on recombination in *H. pylori* employed an antibiotic resistance cassette as selectable marker and thus assessed heterologous recombination. DNA is thought to be single stranded on uptake into the cytoplasm of *H. pylori*^18^, and thus should not be a substrate for cleavage by REases. During the incorporation of fragments of homologous DNA into the chromosome, the intermediate double stranded heteroduplex is hemi-methylated and offers no possibility for REases to cleave. A model has recently been proposed for how REases could attack incoming heterologous sequences and thus limit integration of cassettes flanked by homologous DNA^42^, which seems fully compatible with our data. The integration of cassette-encoded resistance markers flanked by homeologous sequences always goes along with a single-stranded heteroduplex formation during recombination, as a complementary sequence is missing for the heterologous DNA strand. This single-stranded part of the heteroduplex cannot be newly methylated and, on resolution of heteroduplex structure by the replication fork, the complementary strand is newly synthesized. In this time-frame directly following synthesis of complementary sequence, the now double-stranded DNA strand of the incorporated cassette is not methylated. This would enable REases to target possible restriction sites and, thus, could inhibit DNA incorporation during heterologous transformation. This model is in good agreement with our data and would explain why *H. pylori* can show evidence of free recombination^6^,^8^,^9^,^10^,^12^,^14^, while generally conserving gene content and synteny. The observed paucity of imports in the plasticity zones (Fig. 1), regions of the *H. pylori* chromosome with diverse gene content and reduced synteny is in agreement with this hypothesis.

## Methods

### Bacterial strains and culture conditions

*H. pylori* and *Escherichia coli* strains used in this study are listed in Supplementary Table 1. Wild-type and mutant *H. pylori* strains were cultured from frozen stocks on blood-agar plates^7^. For liquid cultures, bacteria were routinely grown in brain heart infusion broth (BHI, BD Difco, Heidelberg, Germany) with yeast extract (2.5 g l^-1^), 10% heat-inactivated horse serum and a mix of antibiotics (vancomycin (10 mg l^-1^), polymyxin B (3.2 mg l^-1^), amphotericin B (4 mg l^-1^), and trimethoprim (5 mg l^-1^)). Cultivation was performed at 175 r.p.m. and 37 °C in microaerobic atmosphere using air-tight jars (Oxoid, Wesel, Germany) and Anaerocult C gas generating bags (Merck, Darmstadt, Germany).

For DNA cloning experiment, *E. coli* strains DH5α, DH5α λpir and MC1061 were cultured in LB or on LB agar plates (Lennox L Broth, Invitrogen–Life Technologies, Darmstadt, Germany) supplemented with ampicillin (200 μg ml^-1^), kanamycin (20 μg ml^-1^) and/or chloramphenicol (20 μg ml^-1^) as required.

### DNA techniques

All molecular biology procedures (cloning, DNA amplification, purification and manipulation) were performed according to standard protocols^43^. Purification of genomic bacterial DNA was carried out using the QIAamp DNA Mini Kit (QIAGEN, Hilden, Germany). High-molecular-weight gDNA required for transformation experiments was prepared using QIAGEN Genomic-tip 100/G and QIAGEN Genomic-tip 500/G (QIAGEN).

### Insertion mutagenesis in *H. pylori*

Mutant strains were constructed by natural transformation-mediated allelic disruption^44^,^45^ of the corresponding target genes^24^. Oligonucleotides used for mutagenesis are provided in Supplementary Table 2. The resulting plasmids (Supplementary Table 3) were used for natural transformation of *H. pylori* strains 26695 and J99-R3. Successful chromosomal replacement of the target gene with the modified allele via allelic exchange was tested by PCR amplifying the relevant loci.

Plasmid pSUS3132 was constructed by overlap PCR^46^. For this, two fragments of ∼500 bp comprising either the N- or C-terminal part of the hp1351 gene and the *aphA-3* cassette were amplified via PCR. By means of oligonucleotide design (Supplementary Table 2), amplification of fragment 1 introduced a PstI restriction site at its 5′-end and a random overlap sequence A at its 3′-end, while a random overlap sequence B and a BamHI restriction site were introduced at the 5′- and 3′-end of fragment 2, respectively. Sequences A and B are complementary to the sequences at the 5′- and 3′-ends of the *aphA-3* cassette, respectively, introduced during amplification. The two fragments and the *aphA-3* cassette were used as templates in the subsequent overlap PCR with the sequences A and B allowing a ligation of the three fragments and with it a continuous amplification of a modified hp1351 allele disrupted by the *aphA-3* cassette. The resulting overlap fragment was ligated into the plasmid pUC19 via PstI and BamHI restriction sites and used for natural transformation of *H. pylori* 26695. As described above, successful chromosomal replacement of hp1351 was tested by PCR.

### Construction of marker-free mutations in *H. pylori*

Multiple target genes were deleted in frame by amplifying ∼1.5 kb of the upstream and downstream region immediately flanking the genes of interest. The oligonucleotides used for mutagenesis (Supplementary Table 2) were designed to (a) introduce a PstI and/or PspOMI restriction site at the 5′-end of the upstream fragment and the 3′-end of the downstream fragment; and (b) to insert random overlap sequences at the 3′-end of the upstream fragment and the 5′-end of the downstream fragment which are complementary to each other. Both fragments were subsequently used as templates in the overlap PCR^46^ with the complementary sequences allowing a continuous amplification of a 3 kb fragment lacking the target gene in the centre. These fragments were ligated into pNPTS138-R6KT, which was either restricted with PstI and PspOMI or PstI or PspOMI as required. To obtain a large amount of DNA for subsequent co-transformations, the resulting plasmids were used as templates for PCR amplification of the overlap fragments. These amplicons were then used for natural co-transformation in *H. pylori* described below.

### Multiplex genome editing in *H. pylori*

Based on a previous study describing MuGENT for *V. cholerae*^32^, we established a protocol for natural co-transformation in *H. pylori* enabling the generation of single and multiple marker-free mutations. For the generation of a Type II REase deletion mutant of *H. pylori* 26695 (Bac555), marker-free alleles lacking the active REase genes hp0053 (HpyAV), hp0091 (HpyAIII), hp1351 (HpyAIV) and hp1366 (HpyAII) were constructed with 1.5 kb homology arms on either side as described above and PCR-amplified (unselected PCR product—no resistance marker). In addition, the *rdxA* allele disrupted by the *aphA-3* or CAT cassette was amplified from pSUS2928 or pCJ535, respectively (0.4 kb homology arms on either side, selected PCR product). For the first co-transformation reaction, *H. pylori* strain 26695 was cultivated on blood-agar plates containing 10% heat-inactivated horse serum and a mix of antibiotics (vancomycin (10 mg l^-1^), polymyxin B (3.2 mg l^-1^), amphotericin B (4 mg l^-1^) and trimethoprim (5 mg l^-1^)). Subsequently, a mixture of 2.5 μg of each of the four unselected and the selected PCR product *rdxA*::CAT was used for natural transformation of 26695. After 24 h of incubation, transformants were selected on blood-agar plates supplemented with chloramphenicol by cultivation for another 72 h. The second co-transformation was initiated by plating a pool of transformants onto standard blood plates described above. Co-transformation was then carried out with a mixture of 2.5 μg of each of the four unselected and the second selected PCR product *rdxA*::*aphA-3*. Transformants were incubated for 24 h and then transferred on blood-agar plates supplemented with kanamycin for selection. Following 72 h of cultivation, a pool of transformants was plated onto standard blood plates to start another round of co-transformation using the *rdxA*::CAT allele for selection. Multiple rounds of co-transformation were performed, which exchanged the resistance cassette in the *rdxA* locus after each round. To screen for the co-transformation frequency, the genotype of ∼20 transformant clones was analysed after every round of co-transformation by PCR amplifying the relevant REase loci. Two 26695 quadruple mutants lacking all four active Type II REase genes were obtained using this protocol; the selection marker in the *rdxA* locus remained in the genome and was used for selection in subsequent experiments. We applied whole-genome sequencing using the Illumina MiSeq platform to confirm the successful marker-free deletion of the REase genes and the absence of non-silent SNPs.

Marker-free deletions of candidate genes involved in the recombination process were also generated via co-transformation of *H. pylori* strain 26695 with one or two unselected alleles lacking the target gene(s) together with one of the two selected PCR products. The PCR products had the same length of homology and the co-transformation was performed exactly as described above.

### TC experiments

Liquid cultures of the *H. pylori* recipient strain 26695 were grown for a time period of 28–52 days (37 °C, 175 r.p.m., microaerobic conditions). In the evening, the cultures were diluted to a cell number of 2.1 × 10^7^ cells per ml and were then grown overnight for ∼14 h to achieve a cell density of ∼3 × 10^8^ cells per ml (corresponding to OD_600_=1). When the appropriate OD_600_ was reached, the recipient cultures were transformed with 2 μg of donor DNA per ml recipient culture and were grown for a further 10 h to start the next TC with dilution of the cultures as described above. As a control, one recipient culture was not transformed. High-molecular-weight gDNA used as donor DNA for transformation was prepared from the J99 derivatives J99-R3 *rdxA*::CAT *flaA*::*aphA-3* (for simplicity referred to as J99-R3^Cm^), as well as the two single MTase mutants either lacking GATC methylation (J99-R3^ΔGATC^) or GANTC methylation (J99-R3^ΔGANTC^) (Supplementary Table 1). In the course of the TC experiments, the recipient cultures were regularly checked for contamination by plating cells onto Columbia agar containing 5% sheep blood (BD Difco). After the terminal cycle, recipient cells were plated onto non-selective blood-agar plates for single colonies. Whole-genome sequencing of randomly chosen single colonies was performed with 454 technology and Illumina MiSeq technology. We chose to sequence 20 genomes of recipient cells transformed with J99-R3^Cm^ to achieve a substantial sample size and compared this with a similar number (18 clones) of recipient cells that were transformed with non-isogenic variants of J99-R3^Cm^ (either J99-R3^ΔGATC^ or J99-R3^ΔGANTC^).

### STT experiments

Liquid cultures of *H. pylori* 26695 and relevant isogenic mutant strains were started with a cell density between 1.8 and 2.1 × 10^7^ cells per ml in the BHI-based medium described above. These pre-cultures were grown for ∼24 h (37 °C, 175 r.p.m., microaerobic conditions) and subsequently used for inoculation of the main cultures starting with a cell density between 1.8 and 2.1 × 10^7^ cells per ml. The main cultures were grown for ∼16 h to achieve a cell density of ∼3 × 10^8^ cells per ml. Five ml of each culture were then transformed with gDNA of the donor strain J99-R3^Cm^ at an excess of 50 genome equivalents per cell. The transformed recipient cultures were subsequently grown for 80 min without shaking (37 °C, microaerobic conditions) to avoid continuing growth but allow selection markers to be recombined and expressed. The cultures were then harvested, resuspended in a defined volume of medium and plated in serial dilutions onto chloramphenicol-containing blood-agar plates for selection. At least two biological replicates were carried out. Single colonies were subjected to whole-genome sequencing using Illumina MiSeq technology. We chose to sequence a total number of 21 genomes to have a comparable sample size as for the TC clones transformed with J99-R3^Cm^.

### *In vitro* transformation system of *H. pylori*

Quantification of recombination rates after natural transformation of *H. pylori* with the transformation systems was performed by transforming *H. pylori* with gDNA of a strain containing a chloramphenicol-resistance cassette within the *rdxA* gene^21^. All experiments were performed in duplicates and reproduced at least two times for each recipient–donor combination. Statistically significant differences were calculated using Welch's *t*-test.

### Whole-genome sequencing

Genome sequencing was performed using 454 FLX Titanium sequencing technology and Illumina MiSeq technology. For 454 pyrosequencing technology, gDNA libraries were prepared according to Genome Sequencer (GS) FLX General Library Preparation Method Manuals for FLX Titanium chemistry or FLX Titanium XL+ chemistry. Emulsion PCR and 454 sequencing on the GS FLX+ instrument (Roche, Mannheim, Germany) were carried out following the manufacturer's instructions. The genomes were sequenced to 22–39 × coverage (Supplementary Table 4). *De novo* assembly of sequence reads was performed with Roche GS De Novo Assembler v2.6 to an average of 64 contigs per strain (±7). Clone-wise preassembly of the contigs with Mauve Contig Mover^47^ was carried out via pairwise alignments against the complete genome sequence of *H. pylori* 26695 (ref. 3) serving as scaffold. Subsequently, multiple alignments of the preassembled contigs of the 26695-derived strains to the published 26695 genome were performed using Mauve v2.3.1 (ref. 48) to generate virtual genomes.

The genomes of most of the TC and all STT clones, as well as the reference strains were sequenced on the Illumina MiSeq platform. Quantitation of sample gDNA was performed using a Picogreen Quant-iT assay (Life Technologies, Darmstadt, Germany). Nextera DNA Sample Preparation Kit (Illumina) was used for gDNA library preparation according to the manufacturer's protocol using 50 ng of gDNA. The fragment lengths were assessed using the Agilent 2100 BioAnalyzer on a High Sensitivity DNA chip and the data used to normalize the samples to a concentration of 4 nmol. The libraries were then loaded onto the MiSeq benchtop sequencer following the protocol for Illumina MiSeq Reagent Kit v3 (600 cycles) and paired-end sequenced (2 × 300 bp) to an expected coverage of 150 × . For analysis, paired-end sequence reads were merged, trimmed and *de novo* assembled using CLC Genomics Workbench 7 (CLC Bio, QIAGEN, Aarhus, Denmark). The Nextera reverse-complement 19 bp adapter (5′-AGATGTGTATAAGAGACAG-3′) was used for trimming of Illumina reads. Per-clone coverages are given in the Supplementary Tables 5 and 6. Contig sets for each sequenced clone were individually arranged against the published genome sequence of 26695 (ref. 3) using Mauve Contig Mover^47^ and subsequently concatenated clone-wise to generate virtual genomes.

### Genome comparisons

First, the genome sequences of the J99-derived donor strains J99-R3^Cm^, J99-R3^ΔGATC^ and J99-R3^ΔGANTC^ were annotated based on the published genome of *H. pylori* J99 (ref. 4) using the Kodon software v3.62 (Applied Maths, Sint-Martens-Latem, Belgium). Following an alignment of these strains to the J99 sequence, a mutation list was exported for every donor strain to precisely locate sequence differences between the isogenic mutants and the parental strain. Insertions and deletions (indels) were excluded from the data set. The same comparative analysis was performed for the re-sequenced *H. pylori* strain 26695 and the publicly available genome sequence of 26695 (ref. 3). Second, the virtual genomes of all transformed clones were annotated and aligned based on the reference genome of *H. pylori* 26695 using Kodon. Subsequently, the virtual genomes of the recipient clones were compared with the respective donor-strain sequence. This enabled exact annotation of *de novo* SNPs and import events including single or clustered nucleotide polymorphisms in the recipient genomes. One to 12 isolated SNPs were detected in the 20 TC- and 21 STT-derived genomes, corresponding to an acquisition of 19.35 SNPs per year (95% confidence interval (CI)=15.09–24.32) and a mutation rate of 1.16 × 10^−5^ per site and year (95% CI=0.91 × 10^−5^–1.46 × 10^−5^).

### Analysis of recombination events

Recombination events were differentiated into single-polymorphism imports (SPIs) and clusters of nucleotide polymorphisms (CNPs). CNPs, by definition, comprise at least two donor-identical polymorphisms that are not separated by >200 bp of recipient-identical sequence^6^, or by a longer stretch (>200 bp) identical to both recipient and donor sequences. However, the latter case occurred rarely throughout the analysis. The detection of SPIs is based on the assumption that small stretches of donor DNA containing only a single polymorphism identical to the donor could have been imported by the recipient strain. However, these single polymorphisms may also derive from *de novo* mutation, which, by chance, introduced the same polymorphism as present in the donor-strain sequence. To get an indication whether donor-identical single polymorphisms derived from mutation or recombination, we analysed the distance between these single polymorphisms to the adjacent polymorphism (on either side) present in the donor sequence. For this, the percentage of abundance for the average lengths of flanking regions of those polymorphisms was plotted and then compared with the values obtained for every single SNP in the J99-R3^Cm^ sequence when compared with 26695 (Supplementary Fig. 7). The flanking lengths of single polymorphisms for both the TC as well as the STT clones were more randomly distributed than compared with all SNP positions in J99-R3^Cm^. This indicated that donor-identical polymorphisms most likely derive from two different origins: mutation and recombination. To determine a cutoff value that allows distinguishing between *de novo* mutations and SPIs, we analysed the flanking lengths of conventional imports (CNPs). Combining the data for all TC and STT clones, the average lengths of flanking regions for all imports ranged between 99 and 106 bp with s.d. ranging between 66 and 69 bp. To set a threshold length for flanking regions of single polymorphisms more likely imported than due to *de novo* mutation, we excluded single polymorphisms that were flanked by donor–recipient identical sequence shorter than the average donor–recipient identical import flanks minus 1 s.d. Based on this, we defined a conservative cutoff value of 40 bp minimal-flanking length, meaning that a donor-identical single polymorphism with a total flanking length >40 bp will be regarded as an SPI in this study. However, it still cannot be excluded with certainty that some of these polymorphisms derived from *de novo* mutation.

With a low frequency, CNPs were interrupted by interspersed sequences of the recipient (ISRs)^21^,^22^. ISRs were set to have 200 bp maximal length and contained single or multiple recipient-identical sites. Sequences identical to the recipient which were longer than 200 bp defined two separate CNPs.

Both CNPs and SPIs were analysed regarding their minimal, median and maximal length (Supplementary Fig. 2a). The minimal length was calculated from the first to the last donor-identical site of a recombined region. The maximal length comprises the minimal import but additionally includes the flanking region to the adjacent donor-specific polymorphisms that are not imported into the recipient genomes. Minimal lengths of recombination events were used for the descriptions of import length, for example, boxplot and Circos plot. Both minimal and maximal import lengths were averaged to calculate the median length for every import. The median length was used to visualize the distribution of import lengths for both *in vitro* and *in vivo* data on a logarithmic scale.

Genes that contained imported sequences were assigned to functional classes of genes^9^,^10^.

### SNP-density analysis

To measure SNP densities on the whole-genome level, near import borders and within recombination events, we conducted a SNP-density search using a 200-bp window with the target position in the centre of the window (Supplementary Fig. 2b). To determine the overall SNP density per 200 bp between the recipient (26695) and donor (J99-R3^Cm^) genome, we applied a sliding window for every position of the 26695 genome counting the number of SNPs compared with the J99-R3^Cm^ genome. In comparison, we used the same 200-bp sliding window to determine the SNP density of 84 randomly selected authentic recombination events in the transformed recipient clones, starting 100 bp upstream of the first imported polymorphism and ending 100 bp downstream of the last imported polymorphism. To specifically determine the SNP density at import borders, we centred both the first and the last polymorphism of the same 84 imports (minimal coordinates) and documented the SNP density in a fixed 200-bp window.

In an additional analysis, we sought to specifically study the distances between the last imported polymorphism and the immediately proximate difference for the same set of authentic imports (Supplementary Fig. 2b, lower left panel). This distance was compared with the distance between the first and the second proximate difference bordering an import.

### Models for recombination tract length

We applied and compared two models for the length of recombinant fragments. The ‘simple model' assumes that recombination event lengths are geometrically distributed. The ‘mixture' model assumes a mixture of Poisson (for the smaller events) and geometric (for the larger events) distributions. The simple model has only a single parameter, which represents the mean length of analysed imports. For the mixture model, there are three parameters corresponding to the mean length for the first Poisson component, the mean length of events of the second geometric component and the mixture proportion between the two distributions. Both AIC (Akaike information criterion) and BIC (Bayesian information criterion) were used to compare the fit of the models in a way that accounts for the greater complexity of the mixture model relative to the simple model.

We also applied the same two models to data from two previous *in vivo* studies: in the first one serial *H. pylori* isolates were obtained at 3–16-year intervals from chronically infected individuals from Columbia and analysed for genome-wide recombination^9^ and in the second one imports were detected in *H. pylori* family isolates^10^. To apply our models to these data, we had to account for the fact that these studies reported minimal recombination tracts, ranging from one recombinant polymorphic site to another, rather than the whole recombinant length which would include non-polymorphic sites on both sides. Based on an average sequence diversity of 5% between unrelated *H. pylori* donor and recipient genomes, the proximate polymorphism of an import was assumed to be 20 bp away from import boundaries. Thus, 20 bp were added to minimal length values to obtain an estimate value.

### Recognition site analysis

To study whether there is correlation between the abundance of recognition sites and borders of imports, a recognition site analysis was performed. To this end, the sequences of all recombined regions were subjected to BLAST search to locate imported sequences on the respective donor chromosomes (J99-R3^Cm^, J99-R3^ΔGATC^ and J99-R3^ΔGANTC^) (Supplementary Fig. 2c). Based on the donor sequence, the distances of the minimal import borders to the next recognition site outside the recombined region—either GATC or GANTC—were determined and then plotted in a histogram.

### Statistical analyses

Statistical analyses were performed using OriginLab 7.5G or GraphPad 6.07. Non-parametric tests were used whenever data were not normally distributed, as indicated in the respective figures.

### Data availability

Sequence data that support the findings of this study have been deposited in the European Nucleotide Archive (ENA) with the study accession numbers PRJEB13893 and PRJEB13894. The authors declare that all other data supporting the findings of this study are available within the article and its Supplementary Information files, or from the corresponding authors upon request.

## Additional information

**How to cite this article:** Bubendorfer, S. *et al*. Genome-wide analysis of chromosomal import patterns after natural transformation of *Helicobacter pylori*. *Nat. Commun.* 7:11995 doi: 10.1038/ncomms11995 (2016).

## Supplementary Material

Supplementary Information Supplementary Figures 1-7, Supplementary Tables 1-6 and Supplementary References

## Figures and Tables

**Table 1:** Import characteristics for recombinant *H. pylori* clones from transformation cycle and short-term transformation experiments.

	26695 transformed with J99-R3^Cm^ gDNA (20 clones)	26695 transformed with J99-R3^ΔGATC^ gDNA (12 clones)	26695 transformed with J99-R3^ΔGANTC^ gDNA (6 clones)
Total no. of CNPs/SPIs	826	547	224
Mean no. of CNPs/SPIs per strain	41.3±24.1	45.6±25.9	37.3±26.7
Range of CNPs/SPIs per strain	4–92	1–92	1–65
Mean total combined length of CNPs/SPIs (minimal) (bp)	55,644.2±36,108.3	58,951.3±34,193.0	56,010.3±38,819.9
Range of total combined lengths of CNPs/SPIs (minimal) (bp)	6,249–128,796	80–116,126	555–108,784
Mean per cent of genome exchanged (minimal) (%)	3.3±2.2	3.5±2.1	3.3±2.3
Range of per cent of genome exchanged (minimal) (%)	0.4–8	0.004–6.9	0.03–6.5
Mean length of CNPs/SPIs (minimal) (bp)	1,347.3±1,586.9	1,293.3±1,370.2	1,500.3±1,624.3
Range of lengths of CNPs/SPIs (minimal) (bp)	1–13,408	1–12,177	1–10,143
Median length of CNPs/SPIs (minimal) (bp)	901.0	964.0	1,007.5
Mean total combined length of CNPs/SPIs (maximal) (bp)	60,226.7±38,158.7	63,840.3±37,022.9	60,210.5±41,712.0
Mean per cent of genome exchanged (maximal) (%)	3.6±2.3	3.8±2.2	3.6±2.5
Mean length of CNPs/SPIs (maximal) (bp)	1,439.4±1,599.6	1,400.5±1,373.8	1,612.8±1,624.8
Median length of CNPs/SPIs (maximal) (bp)	1,003.0	1,077.0	1,139.0

26695 transformed with J99-R3^Cm^ gDNA (21 clones)	Selected	Non-selected	Combined
Total no. of CNPs/SPIs	21	80	101
Mean no. of CNPs/SPIs per strain	1	4.7±4.5	4.8±4.5
Range of CNPs/SPIs per strain	ND	0–20	1–21
Mean total combined length of CNPs/SPIs (minimal) (bp)	ND	4,686.1±4,434.8	6,716.3±5,207.6
Range of total combined lengths of CNPs/SPIs (minimal) (bp)	ND	70–22,297	1680–23,777
Mean length of CNPs/SPIs (minimal) (bp)	2,990.9±1,788.9	995.8±1,158.8	1,410.6±1,537.2
Range of lengths of CNPs/SPIs (minimal) (bp)	638–7,601	1–5,506	1–7,601
Median length of CNPs/SPIs (minimal) (bp)	2,647.0	632.5	934.0
Mean total combined length of CNPs/SPIs (maximal) (bp)	ND	5,136.2±4,777.3	7,272.1±5,528.1
Mean length of CNPs/SPIs (maximal) (bp)	3,195.2±1,625.6	1,090.5±1,175.1	1,528.1±1,543.6
Median length of CNPs/SPIs (maximal) (bp)	2,881.0	709.5	1,098.0

CNP, clusters of nucleotide polymorphism; gDNA, genomic DNA; ND, not determined; SPI, single-polymorphism import.
