# Supplementary material for: Genome-wide analysis of chromosomal import patterns after natural transformation of Helicobacter pylori
Source: Nat Commun. 2016 Jun 22;7:11995. doi: 10.1038/ncomms11995 (PMC4917963; doi:10.1038/ncomms11995)
Supplement: Supplementary Information — Supplementary Figures 1-7, Supplementary Tables 1-6 and Supplementary References [file ncomms11995-s1.pdf]

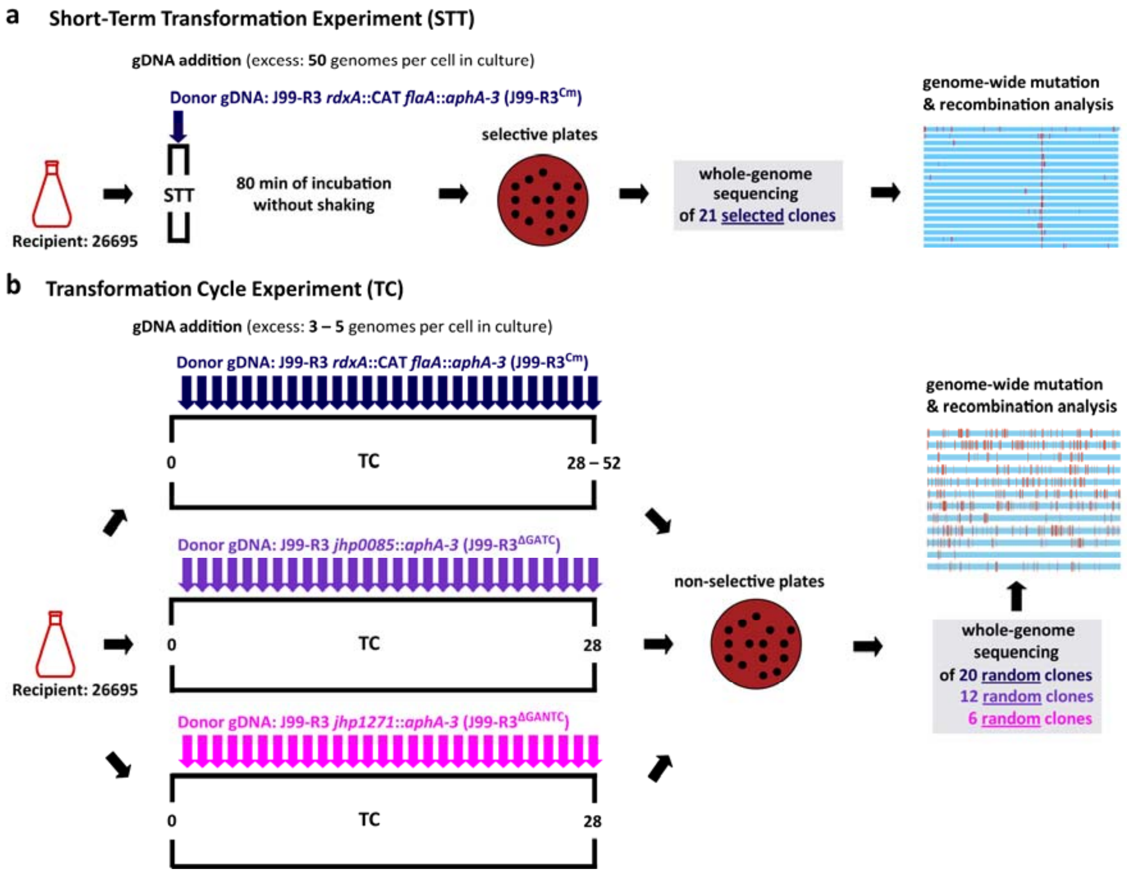

Supplementary Figure 1: Schematic overview of the STT (**a**) and TC (**b**) experimental setup. Color-coding of the donor gDNA is the same as in Fig. 1 and 3. **a**, Liquid cultures of *H. pylori* 26695 were naturally transformed with an excess of 50 genomes of J99-R3^{Cm} donor gDNA per recipient cell. After 80 min of incubation, serial dilutions of the cultures were plated onto Cm-containing blood agar plates for selection of the *rdxA*::CAT allele. Single colonies were randomly selected and their gDNA was subjected to Illumina MiSeq for whole-genome sequencing. The sequences were mapped to the *H. pylori* 26695 genome and subsequently analyzed for genome-wide mutation and recombination events. In the scheme on the right, vertical lines (red) correspond to recombination events in genomes (blue bars) of the STT clones. **b**, Liquid cultures of *H. pylori* 26695 were repeatedly transformed with an excess of 3-5 genomes of donor gDNA per recipient cell over a time period of 28-52 days. Natural transformation was performed individually with gDNA of three different donor strains. Independent recipient cultures were grown for ~14 h before donor DNA was added and following an incubation of 10 h an aliquot of the culture was transferred to fresh medium to start another cycle of transformation. After completion of the final transformation cycle, serial dilutions of the cultures were plated onto non-selective blood agar plates. Single colonies were randomly selected and their gDNA was subjected to 454 pyrosequencing or Illumina MiSeq technology for whole-genome sequencing. The sequences of the TC clones were analyzed as described for the STT experiment.

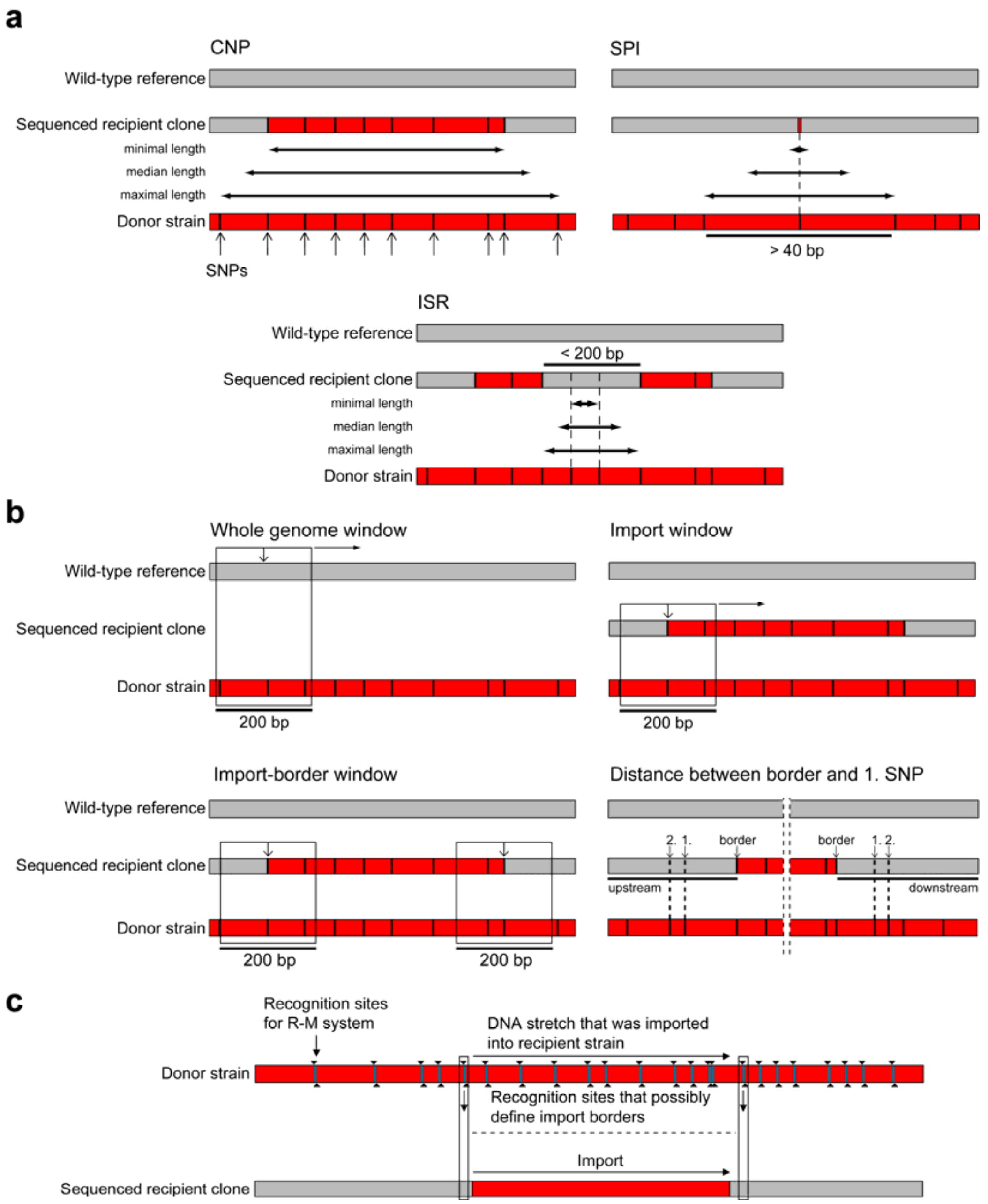

Supplementary Figure 2: Schematic overview of import parameters (**a**), SNP density and distance analyses (**b**), and mapping of restriction sites (**c**). Grey and red horizontal bars represent regions of the *H. pylori* 26695 recipient and the donor strain genome, respectively. Red stretches within the grey bars of recipient clones represent recombination events. **a** and **b**, Black vertical lines indicate positions of sequence polymorphisms between donor and recipient. **c**, Blue vertical lines depict recognition sites for R-M systems and the black triangles represent cleavage sites for REases.

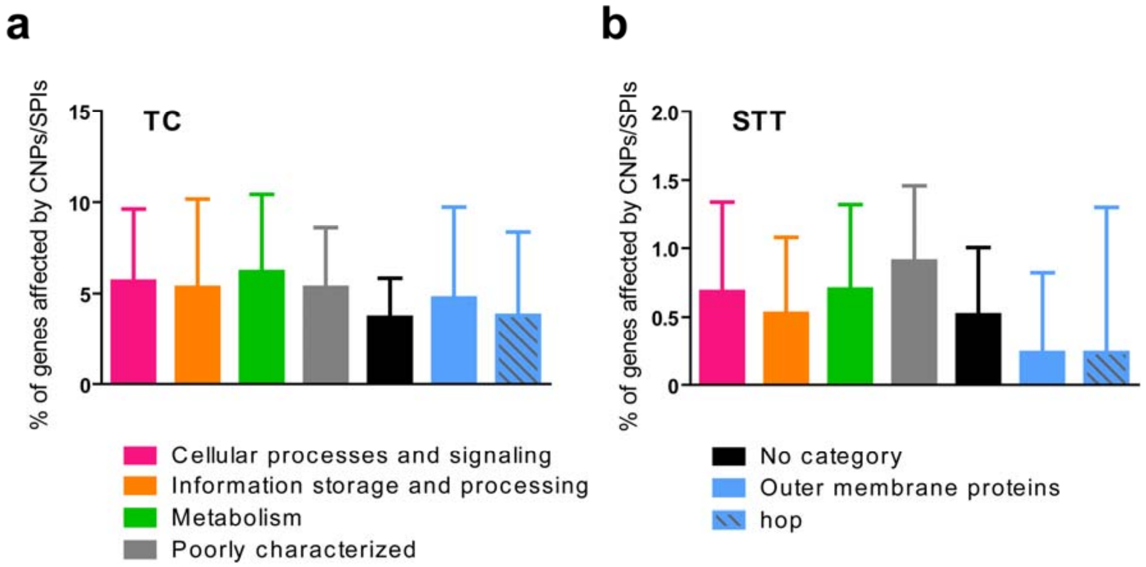

Supplementary Figure 3: Proportion of genes from different functional classes of encoded proteins affected by CNPs/SPIs in the TC (**a**) and the STT (**b**) experiments. Import frequency per category was calculated by normalizing to imports per strain and total genes within functional classes in 26695. Legend displays color-coding of bars. Error bars depict standard deviations. Outer membrane proteins (blue) and hop subfamily (blue with lines) classification is based on ref.[1].

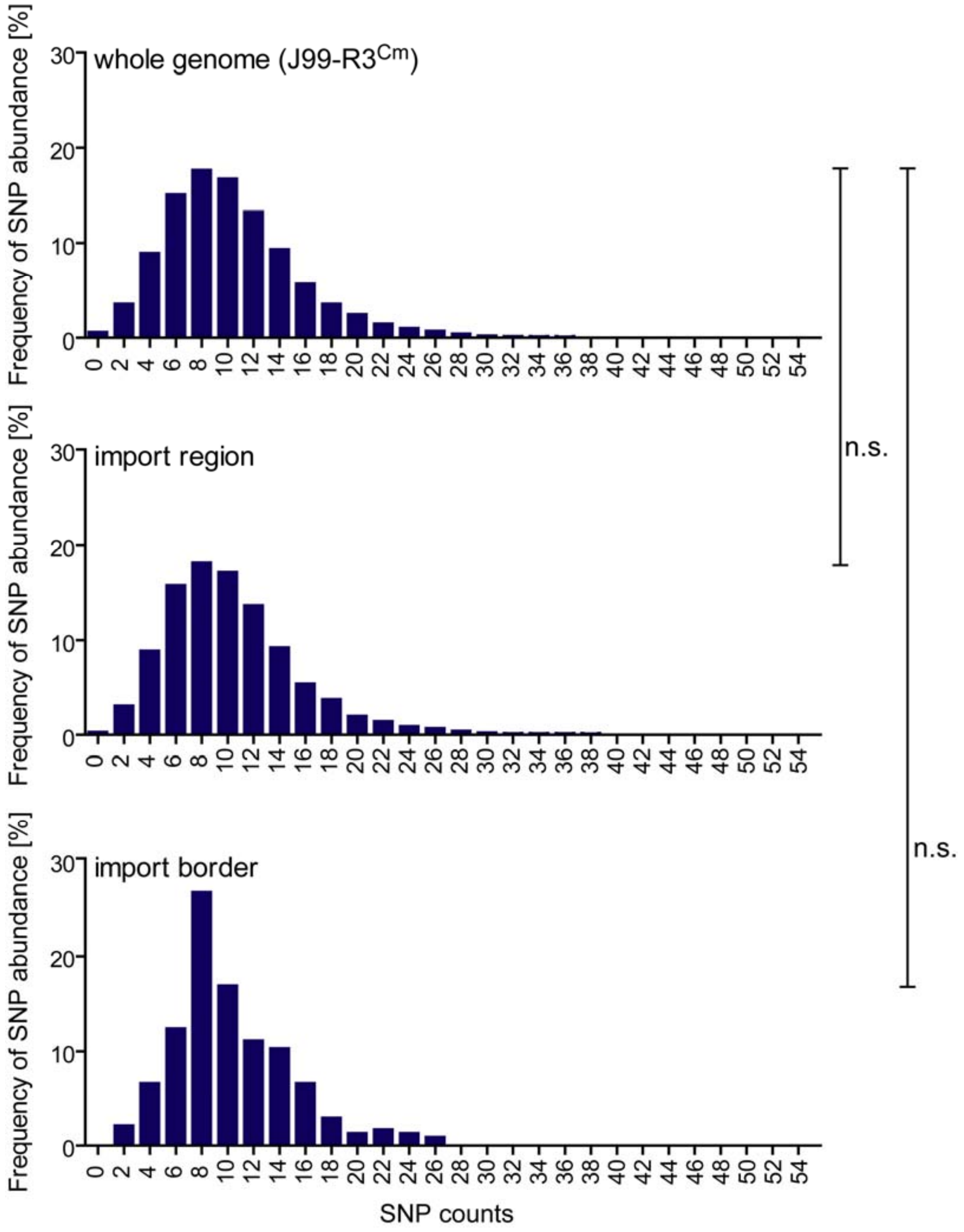

Supplementary Figure 4: SNP density analyses for *H. pylori* 26695 and transformant clones. The relative frequency of the SNP density on the whole-genome level (J99-R3^{Cm}), within recombination events (import region) and near import borders is plotted (see Materials and Methods for details). The number of SNPs within 200-bp windows was determined with the target position in the center of the window. Welch's *t* test (two-sided) was used to determine statistically significant differences. n.s. - not significant.

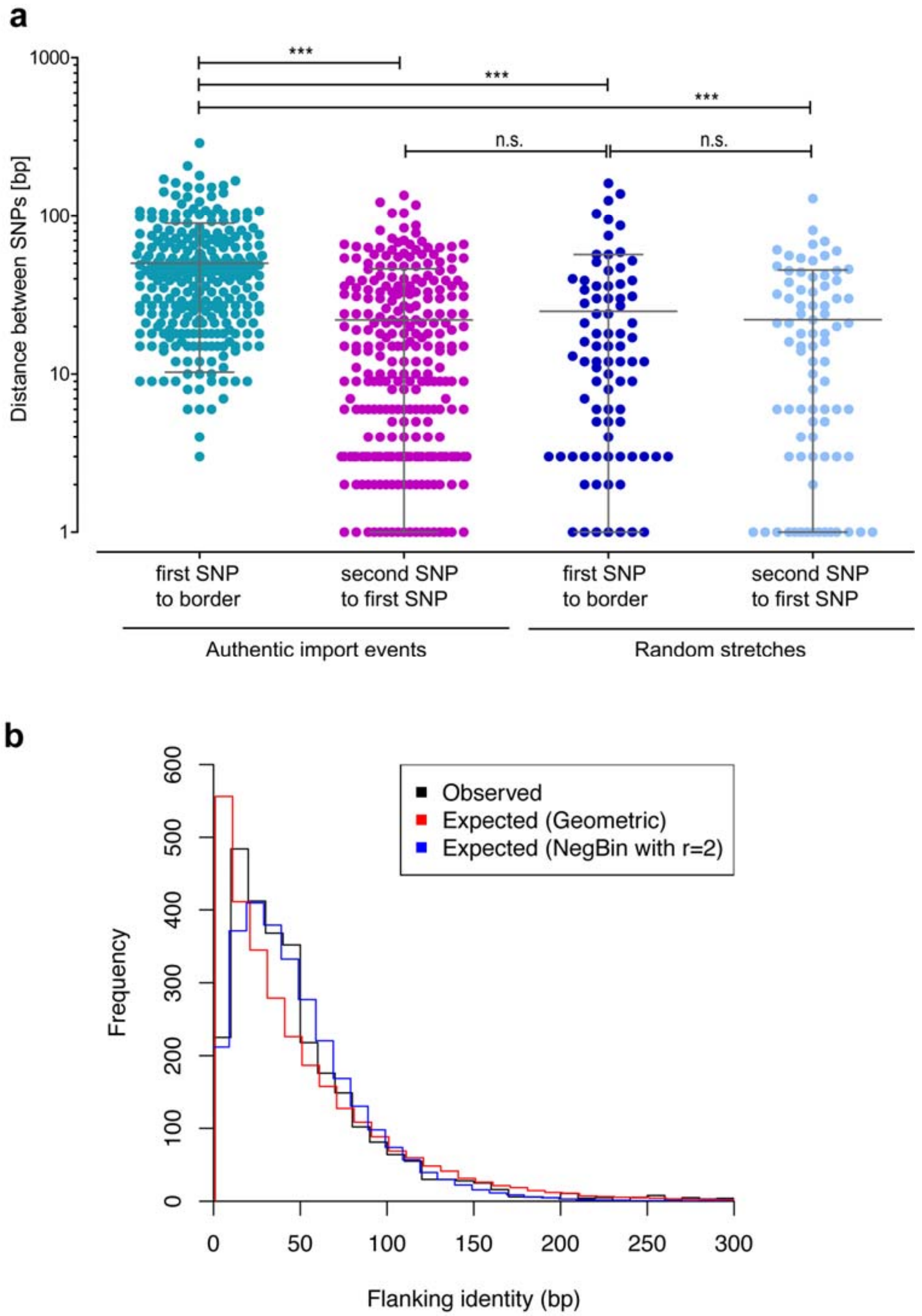

Supplementary Figure 5: a, Scatter plots representing the distance between selected SNPs for the set of 84 representative import events and 40 random stretches used for Figure 3. For each import or random stretch, the distances between the proximate donor-specific polymorphism flanking the import/stretch (first SNP) and the SNP bordering an import/stretch (border) as well as the distance between the second proximate donor-specific polymorphism flanking the import/stretch (second SNP) and the first SNP were plotted on a logarithmic scale. Each dot represents one value. The arithmetic mean is indicated by a long vertical line and the standard deviation by short vertical lines. Significant differences were determined using non-parametric Mann Whitney test (two-sided) and are labeled with asterisks (*** - $p < 0.001$), n.s. - not significant. **b,** Comparison of the observed distribution of length of identical sequence flanking recombination events (black) with expectations under a Geometric model (red) and Negative-Binomial model with parameter r=2 (blue).

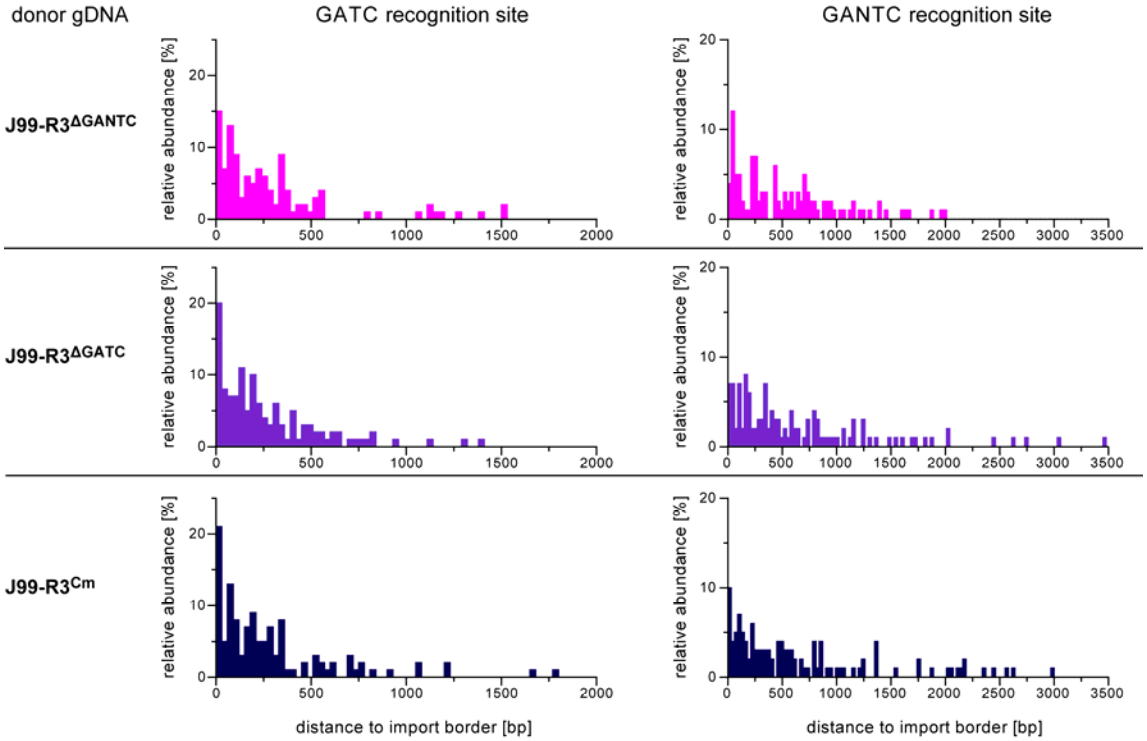

Supplementary Figure 6: Distribution of GATC or GANTC recognition sites in the proximity of import borders for different recipient-donor combinations of TC clones. Strain 26695 was used as recipient and the respective donor gDNA is indicated retaining the same color code as in Fig. 3. The relative abundance of GATC or GANTC recognition sites within the flanking region of approx. 60 randomly selected recombination events per combination were plotted. Non-parametric Mann Whitney test was used assuming Gaussian approximation to compare populations regarding import to GATC-recognition site (left side) or GANTC-recognition site (right side). Populations were not significantly different.

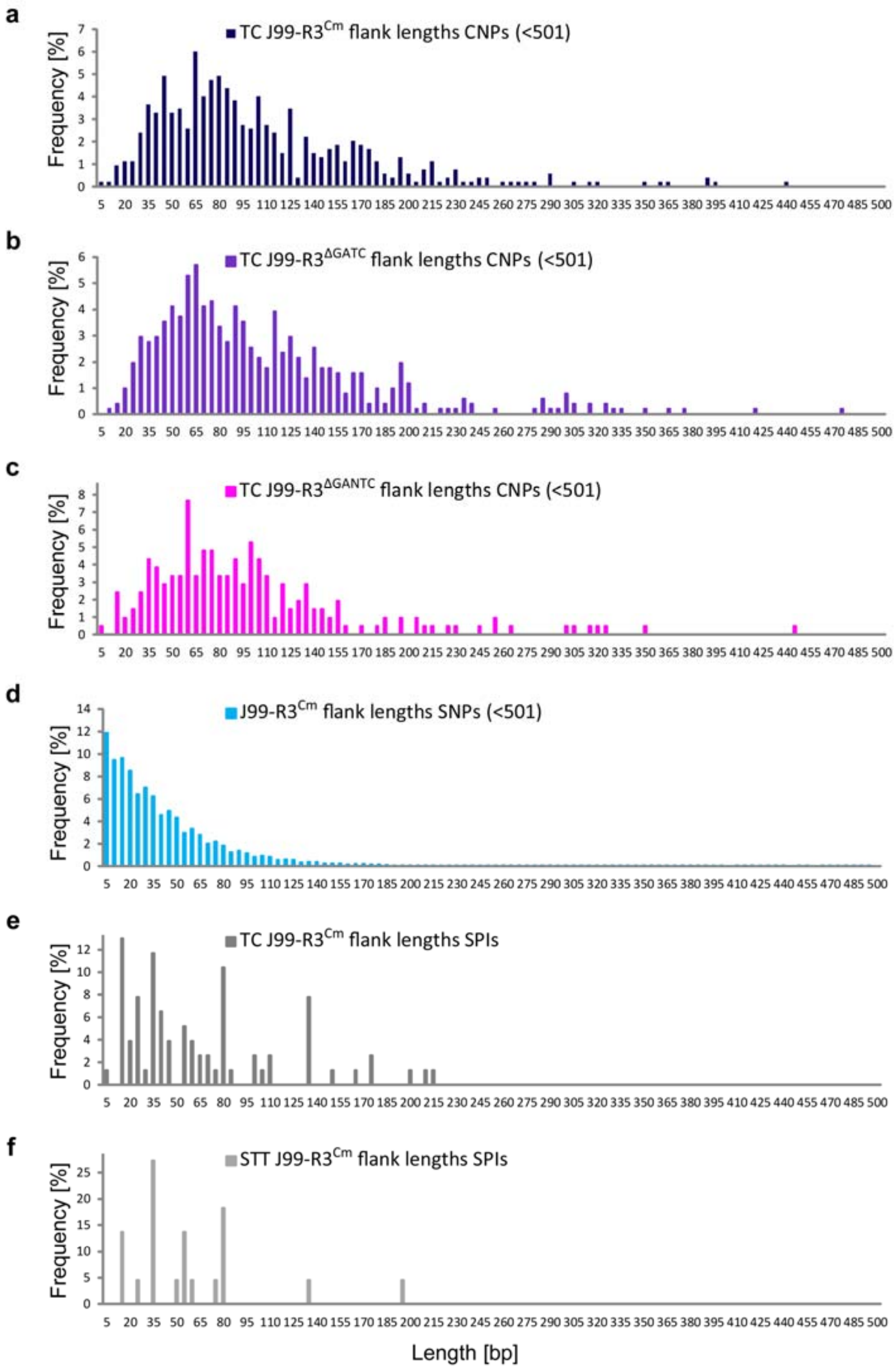

Supplementary Figure 7: Analysis of the length distribution of flanking region for CNPs of TC clones (**a - c**), SNPs between 26695 and J99-R3^{Cm} (**d**) and SPIs of TC and STT clones (**e** and **f**). The relative frequency of events was plotted and the bin size for the length distribution was 5 bp. **a – c,** The lengths of the flanking regions of all import events detected for the TC clones were analyzed. For this, the combined distance of both import borders to the proximate SNP upstream and downstream of a particular import was plotted for every recombination event. Only values below 501 bp were included. **d**, Based on the sequence comparison between *H. pylori* 26695 (recipient) and J99-R3^{Cm} (donor), the size of the donor-recipient identical flanking region for every single SNP was plotted, which comprises the combined distance of a particular SNP to the next SNP in the upstream and in the downstream region. Only values below 501 bp were included. **e** and **f**, The length of flanking regions of the SPIs detected for TC and STT clones was determined as described for panel **d**.

Supplementary Table 1: Bacterial strains.

Strain	Genotype	Source
Escherichia coli		
DH5α	F^-, φ80d*lacZ*ΔM15, Δ(*lacZYA-argF*)U169, *deoR*, *recA*1, *endA*1, *hsdR*17(rk$^-$, mk$^+$), *phoA*, *supE*44, λ^-, *thi*-1, *gyrA*96, *relA*1	[2]
DH5α λpir	ϕ80d*lacZ* ΔM15 Δ(*lacZYA-argF*)U169 *recA1 hsdR17 deoR thi-l supE44 gyrA96 relA1*/λpir	[3]
MC1061	*ara*D139, Δ(*ara*, *leu*)7697, Δ*lac*X74, *galU*$^-$, *galK*$^-$, *hsr*$^-$, *hsm*$^-$, *strA*	[4]
Helicobacter pylori		
26695	*H. pylori* wild-type strain	[5]
Bac339	J99-R3*rdx*::*cat flaA*::*aphA-3* (referred to as J99-R3^{Cm}), derivative of J99-R3	This study
Bac454	J99-R3*jhp0085*::*aphA-3* (referred to as J99-R3$^{\Delta GATC}$), inactivation of the GATC-specific Type II MTase JHP0085 (M.Hpy99VI) from strain J99-R3	[6]
Bac465	J99-R3*jhp1271*::*aphA-3* (referred to as J99-R3$^{\Delta GANTC}$), inactivation of the GANTC-specific Type II MTase JHP1271 (M.Hpy99IX) from strain J99-R3	[6]
Bac534	J99-R3*jhp0085*::*aphA-3 rdxA*::*cat* (referred to as J99-R3$^{\Delta GATC\text{-}Cm}$), derivative of Bac454 containing a *rdxA*::*cat* disruption	This study
Bac535	J99-R3*jhp1271*::*aphA-3 rdxA*::*cat* (referred to as J99-R3$^{\Delta GANTC\text{-}Cm}$), derivative of Bac465 containing a *rdxA*::*cat* disruption	This study
Bac555	26695Δ*hp0053* Δ*hp0091* Δ*hp1351* Δ*hp1366 rdxA*::*aphA-3*, marker-free deletion mutant of the four active Type II REases of 26695	This study
Bac568	J99-R3*jhp0085 jhp1271 rdxA*::CAT (referred to as J99-R3$^{\Delta GATC\ \Delta GANTC}$), J99-R3 containing a *rdxA*::*cat* disruption and marker-free partial deletions of the Type II MTases JHP0085 (M.Hpy99VI, GATC) and JHP1271 (M.Hpy99IX, GANTC) using alleles derived from pSUS3017 and pSUS3020	This study
Bac570	26695*rdxA*::*aphA-3*	This study
Bac569	26695*rdxA*::CAT	This study
J99-R3	Derivative of J99; A to T mutation at position 1618 in *rpoB*; Rif^r	[7]

Supplementary Table 2: Oligonucleotide primers used for *H. pylori* insertion mutagenesis.

Primer	Target gene	5' → 3' sequence*	RS**	Application	Source
HPrdxA-1		TTT AAA TTT GAG CAT GGG GCA G	-	Amplification of *rdxA* locus from pCJ535 and pSUS2928	This study
HPrdxA-2		TGA AAA CAC CCC TAA AAG AGC G	-		This study
PstI-HP0053-up-fwd	hp0053	T GGC CTG CAG GCT GAT GCT TTT AAT GAA TAT CCC T	PstI	In frame deletion of hp0053 (CCTTC-specific Type II REase)	This study
OL-ΔHP0053-rev		*TAG CTT GAA AGC* TTT CAT AAG CTA CTC CTT AAA ATT CCA	-		This study
OL-ΔHP0053-fwd		*TAG CTT ATG AAA* GCT TTC AAG CTA AAA TTT TAA TAA GTT TTA TTG	-		This study
PspOMI-HP0053-dwn-rev		GGA GGG CCC AGT GAT TAG GGC TTG TTT ATC GC	PspOMI		This study
Check-HP0053-fwd		GCC AAA ACA CGA TCC ACC CC	-		This study
Check-HP0053-rev		TTT CGG TTT GAG TGA TGT CCC C	-		This study
PstI-HP0091-up-fwd	hp0091	T GGC CTG CAG AAA CGC CTT TAT GGA AGA CAT TCC	PstI	In frame deletion of hp0091 (GATC-specific Type II REase)	This study
OL-ΔHP0091-up-rev		*AAT GCG CGA TTA* GTT TTT CAT TTT TGC TCC GCT TTA ATG	-		This study
OL-ΔHP0091-dwn-fwd		*AAA ATG AAA AAC* TAA TCG CGC ATT CTA ATG AAA TCG	-		This study
PspOMI-HP0091-dwn-rev		GGA GGG CCC TGA TAG GGT GAG TCA AGA AAT CGT	PspOMI		This study
Check-HP0091-fwd		TTG CCA ATC TTG CTA TAA GCG AC	-		This study
Check-HP0091-rev		AAG CTA GTA AGA GAC GCG CTC	-		This study
PstI-HP1351-up-fwd	hp1351	T GGC CTG CAG ATA TCG CCC TTA TCT TAT CGC G	PstI	In frame deletion of hp1351 (GANTC-specific Type II REase)	This study
OL-ΔHP1351-up-rev		*GAG CCT CTA TCG* GTC CAT TAA GAG TCC TTT TGG CAG	-		This study
OL-ΔHP1351-dwn-fwd		*CTC TTA ATG GAC* CGA TAG AGG CTC TAA AGT AAG CC	-		This study
PspOMI-HP1351-dwn-rev		GGA GGG CCC ATC GTT GAT CAT TTT AGG AGT AAT CTC	PspOMI		This study
Check-HP1351-fwd		AAT CTT GCA AAA TCC ACC CCC C	-		This study
Check-HP1351-rev		GTT TTC AAG CTG TCA ATA GCG GT	-		This study
PstI-HP1366-up-fwd	hp1366	T GGC CTG CAG ATG TTG AAG ATT TAG GGG CGT TAG	PstI	In frame deletion of hp1366 (GAAGA-specific Type II REase)	This study
OL-ΔHP1366-up-rev		*ATT TTA TCA GCC* CAT GTC AAA TAT CCT TTT TTA TTC GCA TT	-		This study
OL-ΔHP1366-dwn-fwd		*ATA TTT GAC ATG* GGC TGA TAA AAT AAA GCG GTG TCT	-		This study
PspOMI-HP1366-dwn-rev		GCA GGG CCC GCG TGT TTT CTT TAA TAA TCA GGG C	PspOMI		This study
Check-HP1366-fwd		ACA AGA TTT ACA GCA TTC AGC CC	-		This study
Check-HP1366-rev		CTT GTG GTA GTC TAT CAT CTC GC	-		This study

*restriction sites are labeled by underlining, overlap regions are highlighted in italics

**RS – restriction site

Supplementary Table 3: Plasmids used in this study.

Plasmids	Genotype	Source
pBHpC8	Cm^r, Source of the *cat* cassette	[8]
pCJ535	Amp^r, Cm^r, pUC19 derivative containing *rdxA* (*hp0954*) disrupted by a *cat* resistance cassette	[9]
pILL600	Amp^r, Km^r, Source of the *aphA-3* cassette	[10]
pNPTS138-R6KT	Km^r, *mob*RP4$^+$ *ori*-R6K *sacB*; beta-galactosidase fragment alpha; suicide plasmid for in-frame deletions or integrations	[11]
pSUS10	Amp^r, Km^r, pHL319-2-4 derivative containing *flaA* from *H. pylori* 898-1 disrupted by the *aphA-3* cassette, source of *flaA*::*aphA3*	[12]
pSUS2928	Amp^r, Km^r, pCJ535 derivative containing *rdxA* (*hp0954*) disrupted by a *aphA-3* resistance cassette	This study
pSUS3017	Amp^r, pUC19 derivative containing jhp0085 lacking a central part of the gene	[6]
pSUS3020	Amp^r, pUC19 derivative containing jhp1271 lacking a central part of the gene	[6]
pSUS3152	Km^r, pNPTS138-R6KT derivative containing up- and downstream regions flanking the Δhp0621 deletion	This study
pSUS3153	Km^r, pNPTS138-R6KT derivative containing up- and downstream regions flanking the Δhp0821 deletion	This study
pSUS3154	Km^r, pNPTS138-R6KT derivative containing up- and downstream regions flanking the Δhp1089 deletion	This study
pSUS3155	Km^r, pNPTS138-R6KT derivative containing up- and downstream regions flanking the Δhp1478 deletion	This study
pSUS3156	Km^r, pNPTS138-R6KT derivative containing up- and downstream regions flanking the Δhp1523 deletion	This study
pSUS3157	Km^r, pNPTS138-R6KT derivative containing up- and downstream regions flanking the Δhp1553 deletion	This study
pSUS3225	Km^r, pNPTS138-R6KT derivative containing up- and downstream regions flanking the Δhp0053 deletion	This study
pSUS3226	Km^r, pNPTS138-R6KT derivative containing up- and downstream regions flanking the Δhp0091 deletion	This study
pSUS3227	Km^r, pNPTS138-R6KT derivative containing up- and downstream regions flanking the Δhp1351 deletion	This study
pSUS3228	Km^r, pNPTS138-R6KT derivative containing up- and downstream regions flanking the Δhp1366 deletion	This study
pUC19	Amp^r, Colx101, MCS within *lacZ*: blue/white selection	[13]

Supplementary Table 4: Genome sequencing coverages for clones from Transformation Cycle (TC) experiments (454 FLX pyrosequencing).

Sample ID	Project	DNA addition	Coverage
Control 1	TC	-	30
Control 2	TC	-	33
1_1	TC	+	35
1_5	TC	+	35
1_9	TC	+	29
1_10	TC	+	31
2_1	TC	+	36
2_5	TC	+	39
2_8	TC	+	22
2_10	TC	+	36

Supplementary Table 5: Genome sequencing coverages for reference strains and clones from Transformation Cycle (TC) experiments (Illumina MiSeq).

Sample ID	Project	Index 1 (I7)	Index 2 (I5)	% Reads Identified (PF)	No. Reads (paired)	Calculated Coverage
References						
H. pylori-26695	reference	CGAGGCTG	CTCTCTAT	1.9222	540,621	101
BAC-454	reference	GTAGAGGA	AGAGTAGA	1.6389	460,942	86
BAC-465	reference	TAAGGCGA	GTAAGGAG	1.8728	526,722	99
BAC-339	reference	CGTACTAG	ACTGCATA	1.8083	508,591	95
TC clones						
1-1-1	TC	TAAGGCGA	TAGATCGC	0.9053	301,044	56
1-1-2	TC	TAAGGCGA	CTCTCTAT	1.8733	622,954	117
1-1-3	TC	TAAGGCGA	TATCCTCT	0.9676	321,778	60
1-1-4	TC	TAAGGCGA	AGAGTAGA	1.1483	381,864	72
1-1-5	TC	TAAGGCGA	GTAAGGAG	1.0900	362,466	68
1-1-6	TC	CGTACTAG	ACTGCATA	1.7822	592,656	111
1-2-1	TC	CGTACTAG	AAGGAGTA	1.4415	479,366	90
1-2-2	TC	AGGCAGAA	AAGGAGTA	1.9520	548,995	103
1-2-3	TC	CGTACTAG	TAGATCGC	0.8129	270,336	51
1-2-4	TC	CGTACTAG	CTCTCTAT	0.8834	293,764	55
1-2-5	TC	AGGCAGAA	TATCCTCT	1.0718	356,420	67
1-2-6	TC	AGGCAGAA	AGAGTAGA	0.8715	289,804	54
2-1-1	TC	AGGCAGAA	GTAAGGAG	1.0266	341,402	64
2-1-2	TC	AGGCAGAA	ACTGCATA	0.8756	291,164	55
2-1-3	TC	AGGCAGAA	AAGGAGTA	0.9076	301,834	57
2-1-4	TC	TCCTGAGC	CTAAGCCT	0.9231	306,988	58
2-1-5	TC	TCCTGAGC	TAGATCGC	0.7762	258,136	48
2-1-6	TC	TCCTGAGC	CTCTCTAT	1.1398	379,026	71
2-2-1	TC	TCCTGAGC	TATCCTCT	1.4702	488,910	92
2-2-2	TC	TCCTGAGC	AGAGTAGA	1.2260	407,712	76
2-2-3	TC	GGACTCCT	GTAAGGAG	1.4386	478,416	90
2-2-4	TC	GGACTCCT	ACTGCATA	1.4043	467,006	88
2-2-5	TC	GGACTCCT	AAGGAGTA	1.2592	418,752	79
2-2-6	TC	GGACTCCT	CTAAGCCT	2.0373	677,506	127
3-1	TC	GGACTCCT	TAGATCGC	1.0747	381,260	71
3-2	TC	TAGGCATG	CTCTCTAT	1.3148	437,236	82
3-3	TC	TAGGCATG	TATCCTCT	1.1417	379,680	71
3-4	TC	TAGGCATG	AGAGTAGA	1.6861	560,714	105
3-5	TC	TAGGCATG	GTAAGGAG	1.7418	579,240	109
3-6	TC	TAGGCATG	ACTGCATA	1.4093	468,672	88

Supplementary Table 6: Genome sequencing coverages for clones from Short Term Transformation (STT) experiments (Illumina MiSeq).

Sample ID	Project	Index 1 (I7)	Index 2 (I5)	% Reads Identified (PF)	No. Reads (paired)	Calculated Coverage
1-1-1-Cm	STT	TAAGGCGA	TAGATCGC	0.6309	379,240	71
1-1-2-Cm	STT	CGTACTAG	CTCTCTAT	18.9819	11,410,908	2140
1-1-3-Cm	STT	AGGCAGAA	CTCTCTAT	1.776	1,067,612	200
1-1-4-Cm	STT	TCCTGAGC	AGAGTAGA	1.9418	1,167,304	219
1-1-5-Cm	STT	GGACTCCT	GTAAGGAG	2.2741	1,367,068	256
1-1-10-Cm	STT	GTAGAGGA	CTCTCTAT	2.3046	1,019,328	191
1-1-11-Cm	STT	GTAGAGGA	TATCCTCT	1.931	854,056	160
1-1-12-Cm	STT	GTAGAGGA	AGAGTAGA	1.4854	657,002	123
1-1-13-Cm	STT	AAGAGGCA	GTAAGGAG	1.1962	529,092	99
1-1-14-Cm	STT	AAGAGGCA	CTCTCTAT	1.8391	813,402	153
1-1-15-Cm	STT	CGAGGCTG	TATCCTCT	2.2141	979,282	184
1-2-1-Cm	STT	CGAGGCTG	AAGGAGTA	1.1986	720,540	135
1-2-2-Cm	STT	AAGAGGCA	ACTGCATA	1.738	1,044,796	196
1-2-3-Cm	STT	GTAGAGGA	GTAAGGAG	2.3832	1,432,668	269
1-2-4-Cm	STT	TAAGGCGA	TAGATCGC	1.8022	835,090	157
1-2-10-Cm	STT	CGAGGCTG	AGAGTAGA	1.985	877,968	165
1-2-11-Cm	STT	GCTACGCT	GTAAGGAG	2.5521	1,128,756	212
1-2-12-Cm	STT	GCTACGCT	CTCTCTAT	2.7786	1,228,970	230
1-2-13-Cm	STT	CAGAGAGG	TATCCTCT	2.0648	913,264	171
1-2-14-Cm	STT	CAGAGAGG	AGAGTAGA	2.2108	977,804	183
1-2-15-Cm	STT	CTCTCTAC	GTAAGGAG	1.5898	703,160	132

Reference List

1. Alm, R. A., *et al.* "Comparative genomics of *Helicobacter pylori*: analysis of the outer membrane protein families," *Infect Immun* **68**, 4155 (2000)

2. Hanahan, D. "Studies on Transformation of *Escherichia coli* With Plasmids," *J Mol Biol* **166**, 557 (1983)

3. Miller, V. L. and Mekalanos, J. J. "A novel suicide vector and its use in construction of insertion mutations: osmoregulation of outer membrane proteins and virulence determinants in *Vibrio cholerae* requires *toxR*," *J Bacteriol* **170**, 2575 (1988)

4. Casadaban, M. J. and Cohen, S. N. "Analysis of Gene Control Signals by DNA Fusion and Cloning in *Escherichia coli*," *J Mol Biol* **138**, 179 (1980)

5. Tomb, J. F., *et al.* "The complete genome sequence of the gastric pathogen *Helicobacter pylori*," *Nature* **388**, 539 (1997)

6. Krebes, J., *et al.* "The complex methylome of the human gastric pathogen *Helicobacter pylori*," *Nucleic Acids Res* **42**, 2415 (2014)

7. Kulick, S., *et al.* "Mosaic DNA imports with interspersions of recipient sequence after natural transformation of *Helicobacter pylori*," **3**, e3797 (2008)

8. Ge, Z., Hiratsuka, K., and Taylor, D. E. "Nucleotide Sequence and Mutational Analysis Indicate That Two *Helicobacter pylori* Genes Encode a P-Type ATPase and a Cation-Binding Protein Associated With Copper Transport," *Mol Microbiol* **15**, 97 (1995)

9. Behrens, W., *et al.* "Role of Energy Sensor TlpD of *Helicobacter pylori* in Gerbil Colonization and Genome Analyses After Adaptation in the Gerbil," *Infect Immun* **81**, 3534 (2013)

10. Labigne-Roussel, A., Courcoux, P., and Tompkins, L. "Gene Disruption and Replacement as a Feasible Approach for Mutagenesis of *Campylobacter jejuni*," *J Bacteriol* **170**, 1704 (1988)

11. Lassak, J., Henche, A. L., Binnenkade, L., and Thormann, K. M. "ArcS, the cognate sensor kinase in an atypical Arc system of *Shewanella oneidensis* MR-1," *Appl Environ Microbiol* **76**, 3263 (2010)

12. Suerbaum, S., Josenhans, C., and Labigne, A. "Cloning and genetic characterization of the *Helicobacter pylori* and *Helicobacter mustelae flaB* flagellin

genes and construction of *H. pylori flaA*- and *flaB*-negative mutants by electroporation-mediated allelic exchange," *J Bacteriol* **175**, 3278 (1993)

13. Yanisch-Perron, C., Vieira, J., and Messing, J. "Improved M13 Phage Cloning Vectors and Host Strains: Nucleotide Sequences of the M13mp18 and pUC19 Vectors," *Gene* **33**, 103 (1985)
